# Do fossil fuel firms reframe online climate and sustainability communication? A data-driven analysis

**DOI:** 10.1038/s44168-023-00086-x

**Published:** 2023-12-18

**Authors:** Ramit Debnath, Danny Ebanks, Kamiar Mohaddes, Thomas Roulet, R. Michael Alvarez

**Affiliations:** 1 University of Cambridge, Cambridge, CB2 1TN UK; 2 California Institute of Technology, Pasadena, CA 91125 USA; 3 Harvard University, Cambridge, MA 02138 USA

**Keywords:** Psychology and behaviour, Business and industry

## Abstract

Identifying drivers of climate misinformation on social media is crucial to climate action. Misinformation comes in various forms; however, subtler strategies, such as emphasizing favorable interpretations of events or data or reframing conversations to fit preferred narratives, have received little attention. This data-driven paper examines online climate and sustainability communication behavior over 7 years (2014–2021) across three influential stakeholder groups consisting of eight fossil fuel firms (industry), 14 non-governmental organizations (NGOs), and eight inter-governmental organizations (IGOs). We examine historical Twitter interaction data (*n* = 668,826) using machine learning-driven joint-sentiment topic modeling and vector autoregression to measure online interactions and influences amongst these groups. We report three key findings. First, we find that the stakeholders in our sample are responsive to one another online, especially over topics in their respective areas of domain expertise. Second, the industry is more likely to respond to IGOs’ and NGOs’ online messaging changes, especially regarding environmental justice and climate action topics. The fossil fuel industry is more likely to discuss public relations, advertising, and corporate sustainability topics. Third, we find that climate change-driven extreme weather events and stock market performance do not significantly affect the patterns of communication among these firms and organizations. In conclusion, we provide a data-driven foundation for understanding the influence of powerful stakeholder groups on shaping the online climate and sustainability information ecosystem around climate change.

## Introduction

The Intergovernmental Panel on Climate Change (IPCC), in the latest Sixth Assessment Report (AR6), calls for immediate climate action^1^. However, this report asserts that a significant barrier to tackling the climate crisis is rampant misinformation on social media^1–3^. This is important, as globally, more than 4 billion people use social media platforms for communication, including information on climate action^2,4^. Climate change misinformation on social media can be crude and direct, for example, spreading knowingly false and misleading information^5^. Misinformation on social media can also be more subtle and indirect; for example, corporate greenwashing is a tactic used to persuade audiences that business practices and products meet green standards while obfuscating the negative environmental performance of a company^6^. Fossil fuel firms are often accused of such obfuscation on social media platforms^7,8^.

In this paper, we study subtle misinformation and conversation reframing akin to greenwashing that allows the fossil fuel industry to influence the communication of climate watchdog organizations on social media. While this study is data-driven and observational, we show that the world’s top polluting fossil fuel firms engage in redirecting and reframing the social media conversation in the broader information ecosystem. This ecosystem involves the primary intergovernmental organizations (IGOs) and non-governmental organizations (NGOs) that operate in the climate and sustainability space. While it is not surprising that subtle forms of climate misinformation like redirection and reframing exist, it is surprising to see that the primary watchdogs for climate misinformation (IGOs and NGOs) may also be subject to influence by the fossil fuel industry.

In the growing climate accountability literature, there is increasing evidence suggesting that climate communications through social media may rely on issue framing that can shift the conversation to align with particular political or corporate frames^6,9–12^. However, previous research does not adequately explore the more subtle role that top fossil fuel firms may play in propagating misdirected communication and how this may affect the broader climate communication ecosystem. When corporate reframing or redirection on social media occurs, how are other significant actors in the climate conversation influenced, especially non-governmental organizations and intergovernmental organizations? Furthermore, how do NGOs and IGOs respond (if they respond) to the fossil fuel industry reframing social media conversations? Our research investigates the communication interactions on social media between the members of the fossil fuel industry and the NGOs and IGOs that focus on climate change and sustainability. We empirically unpack their reframing and redirecting approaches.

Recent research examines how the top fossil fuel firms use social media for greenwashing purposes^13–15^. Much of this work centers on how these firms use social media to generate climate disinformation “echo chambers” (where the same communication agenda is reinforced through the interactions between different stakeholder groups) or “filter bubbles” (where information is selected and highlighted), leading consumers to believe a specific (and firm-driven) discourse. This echo chamber effect emerges through communication triangulation between the three-core stakeholder groups^5,6,14,16–20^. In the literature, echo chambers and filter bubbles are usually studied at an individual level. Still, a significant gap remains in analyzing the collective behavior of stakeholder groups. For example, during the two weeks of the United Nations Conference of Parties-26 (COP-26), research found that 16 of the world’s most polluting fossil fuel companies were associated with more than 1700 climate misinformation ads on Facebook alone, with 150 million user interactions, demonstrating the virality of climate misinformation^14,21^.

Research suggests that fossil fuel firms use a variety of messages emphasizing the idea that consumer behavior is the leading cause of climate change^7,13,22^. Furthermore, these firms are more likely to discuss decarbonization and clean energy in their annual reports as green pledges rather than concrete actions. Their investment portfolio often does not match their climate rhetoric, which provides evidence of greenwashing and potential reframing behavior^8^.

Increasingly, social media platforms also influence climate activism in ways that can create disinformation echo chambers through environmental extremism^5,23–25^. These echo chambers fuel climate action polarization as reframed, and misdirected climate narratives influence broader audiences^5,26^. These actions encourage climate change denialism and fuel organized misinformation campaigns at a systems-scale^6,27–29^. With limited research in this area, it is difficult to know who is involved in online climate misinformation and its underlying communication structure at a systems-level^3,20,28^ and consider its potential neutralization^30,31^. This may undermine public risk judgments about the seriousness of issues like online greenwashing, fake news, conspiracy theories, or radical climate action. Understanding the structure of social media communications between fossil fuel firms, NGOs, and IGOs is a necessary first step before we can decide whether to develop possible measures, like regulatory and policy design changes, self-regulation, or community engagement, to name a few, to counter online misinformation^5,20,31–35^.

## Theoretical framing

We propose a simple theory to explain why key climate stakeholders engage in discussion with each other and how their incentives motivate their behavior. In our theoretical framework, climate stakeholders influence the discussion and shape the bounds of policy debate around climate as a means of achieving their goals. For example, the incentives of fossil fuel firms include protecting their profits and avoiding heavy regulation by demonstrating to policymakers and the public that they engage in sustainable behaviors (supporting^13,15^). IGOs have political incentives, including promulgating policies that sustain the environment and which may mitigate the effects of climate change, while considering the political preferences of the public, who might be more skeptical of climate action.

NGOs aim to reduce climate change, even at the cost of firm profits and political popularity. Each of these stakeholders will reframe public debates to accomplish their goals. Fossil fuel firms have incentives to highlight their sustainability efforts to appease international regulators while trying to maintain profits from their fossil fuel activities. IGOs have incentives to inform the public about the importance of climate policy and emphasize their success in encouraging firms to engage in more sustainable behaviors. NGOs have incentives to both inform the public about climate science and raise awareness of perceived policy failures in the surrounding area to punish political actors they view as moving too slowly and firms they view as purely profit-seeking^36,37^. Thus, reframing the social media discussion is a way for IGOs and NGOs to set terms of debate favorable to their political and climate goals, which are at odds with the goals of the fossil fuel industry. At the same time, the fossil fuel industry could reframe the discussion to focus the online climate debate on their sustainability efforts.

To deepen our understanding of the reframing of social media climate conversations, we also examine contextual factors that might influence online discussion among these agents. These climate-stakeholder firms and groups may be communicating online in response to other factors. That is, they may not be engaged in a joint conversation but instead could be focused on framing organizational reactions to extreme climate events or, in the case of fossil fuel firms, their financial or corporate successes. We aim to describe the extent to which these outside factors influence discussion as opposed to fossil fuel companies reframing the discussion themselves. These external factors are important potential confounding factors in our theory of online conversations between the fossil fuel industry, NGOs, and IGOs, and thus we must account for their potential influences on the interactions between them.

Thus, an important part of our analysis is to include external factors like industry financial returns and extreme climate events in our models. This will allow us to account for these potential confounding factors and test hypotheses regarding whether external events are more of a focus in the online communications of these climate stakeholder firms and groups.

Regarding industry financial returns, there has been past research using social media data like Twitter in models predicting financial systems^38^. As an exogenous factor, Bollen, Mao, and Zeng^39^ have shown that different dimensions of user moods on Twitter can predict the Dow Jones Industrial Average performance. Similarly, Oliveira, Cortez, and Areal^40^ found that Twitter sentiment and posting volume were relevant for forecasting the returns of the S&P 500 index, portfolios of lower market capitalization, and some industries. Thus, we will include measures of the financial market performance of the fossil fuel industry in our analyses below.

Similarly, climate change effects, like extreme temperature anomalies and heat waves, have been found to influence the rate of tweeting amongst people living in the US^41^. However, researchers stress that there is a strong need to develop a better understanding of how actors like NGOs, IGOs, governments, and the private sector in the climate governance space engage on Twitter, interact with the public, and are themselves shaped by such interactions. Such understanding of climate debates on Twitter can inform climate governance research and advance theory on how social media, through norm diffusion, opinion leadership, and citizen and elite opinion formation, impacts climate governance^42^. To account for the potential influence of climate events on the conversations between fossil fuel firms, IGOs, and NGOs, our dynamic models will incorporate information about extreme climate events.

In doing so, we conceptualize that reframing can be measured through interactions between these three groups on social media platforms. Online communication from core stakeholder groups (fossil fuel industry, IGOs, and NGOs) is vital to influencing the public discourse on sustainability and climate change. This paper empirically captures the degree of reframing of online climate communication between fossil fuel firms, IGOs, and NGOs. We predict how stakeholders will change their tendency to discuss a topic online if one group increases their messaging. Our data-driven research design uses a large sample of tweets, retweets, and replies (*n* = 668,826, 2014–2021) from eight of the largest carbon-emitting firms (see Table 1 for information about the fossil fuel firms included in our study)^43^, and the 14 NGOs and eight IGOs with a cumulative follower base of 9.6 million users worldwide (see the section “Data and method” and Supplementary Section 1). Furthermore, we assumed Twitter accounts were influential if they had at least 10,000 followers. Therefore, we only selected the public Twitter accounts of these firms and organizations that matched this criterion (see Supplementary Table 1).

**Table 1:** Cumulative emissions of private fossil fuel firms (1965–2018) in MtCO_2_e (million tonnes of carbon dioxide equivalent) and its global Twitter followers (until September 2021) (Source: Ref. ^43^)

Fossil firm	Emissions (MtCO_2_e)	Followers (until Sept 2021)
Chevron, USA	43,787	374,800
ExxonMobil, USA	42,484	328,100
BP, UK	34,564	106,700
Shell, The Netherlands	32,498	566,200
Peabody Energy, USA	15,783	8700
ConocoPhillips, USA	15,422	163,600
Total SA, France	12,755	16,000
BHP, Australia	10,068	56,100
Total	207,361	1,620,200

Given our theoretical framework, we derive three key hypotheses.

**H1:** We hypothesize that stakeholders will have more ability to direct conversation over topics in their areas of domain expertise. In our case, we expect NGOs and fossil fuel firms to respond to IGOs on policy; that IGOs and fossil fuel firms will react to NGOs on climate action and environmental justice topics; and that NGOs and IGOs will respond to fossil fuel firms on corporate sustainability initiatives.

**H2:** We hypothesize that fossil fuel firms will be more successful at redirecting the online conversation than intergovernmental or nongovernmental groups.

**H3:** We hypothesize that the online communication behavior of fossil fuel firms will be influenced by exogenous factors like extreme weather events and stock market performance.

## Results

### Structure and mapping of stakeholders’ online climate communication

This section presents the results from our joint topic-sentiment (JST) modeling, which provides the foundation for understanding how information flows between the three stakeholder groups in our analysis. To measure both topical structure and sentiment orientation, we use JST modeling to uncover and measure these latent characteristics in the text data. We needed such a method to better capture the context of the uncovered topics—for example, while both corporate sustainability and climate action might have some shared tokens, JST can distinguish between the two by accounting for sentiment, an additional, relevant latent layer in our setting. For example, we show the top-15 tweets from the fossil fuel firms with the highest positive and negative sentiments in Table 2 (the estimation approach is discussed in the “Data and method” section).

**Table 2:** Illustrative tweets from fossil fuel firms and their estimated sentiment scores.

Tweets with highest positive sentiment scores	Tweets with highest negative sentiment scores	Firm name (username)
Congrats to Indigenous School Award winner Gordonvale State High, recognizing celebrating achievements in #STEM	We are deeply sorry to everyone who has and will suffer from this terrible tragedy: CEO Andrew Mackenzie	BHP (@bhp)
Read how our Iron Ore team are helping ecologists understand more about one of Australia’s most unique bat species	As with most issues in China, there are many facets to the debt liability story	BHP (@bhp)
Hi Andi, we’re working to make all types of energy cleaner better from renewable energy cleaner-burning natural gas to advanced fuels new low-carbon businesses, we’re committed to playing out part to advance a love carbon future provide the energy the world needs	I am really sorry you had a bad experience at a BP-branded site. We will raise this unacceptable behavior with the dealer	BP (@bp_plc)
Affordable, reliable #energy is cornerstone of #development helps improve quality of life for communities -Chevron VP Steve Green #CSISGDF	Anti- #Chevron activists recruit phony paid protesters to picket Chevron annual shareholder meeting	Chevron (@Chevron)
We are very proud to support @techbridgegirls @ChabotSpace to encourage curiosity & interest in #STEM #education in young women #EBWC #IWD	Chevron fights to prevent #malaria among the most vulnerable in #Angola pregnant women and children under 5	Chevron (@Chevron)
Lets help bolster the economy with lower, more stable #natgas prices Learn more about #natgas economic benefits:	“Fracking fears unfounded” says retired geologist #natgas	Conoco Phillips (@conocophillips)
The #ConocoPhillips SPIRIT of Conservation program is seeking grant proposals to conserve breeding, stopover and wintering habitats for #migratorybirds, and to provide technical assistance to improve bird habitat conservation practices on working lands	From an Indiana pipeliner: @WhiteHouse rejects #KeystoneXL Sad day for U.S. worker	Conoco Phillips (@conocophillips)
With our research capabilities and commitment to innovation, we are researching new technologies to provide more affordable, lower-carbon energy, Chairman CEO Darren Woods #XOMAnnualMeeting	@foe_us Allegations we deceived the public on #climatechange are misleading and baseless	ExxonMobil (@exxonmobil)
ExxonMobil Colombia helps promote non-violence, gender equality good behavior among youth #IYD2014	Anti-oil activists drummed up false allegations against @exxonmobil #Exxonknew	ExxonMobil (@exxonmobil)
RT @WorldCoal: #Steel is fundamental to a more #sustainable world, helping to build lighter, more efficient vehicles, new highly efficient	Without coal, American families will feel more pain at the plug, akin to pain at the pump.	Peabody (@peabodyenergy)
@PeabodyEnergy proudly supports @ArchGrants toward a more robust #STL #startup culture	RT @NationalMining: Coal’s decline could mean more power shortages #countoncoal #powergrid #economy	Peabody (@peabodyenergy)
We are partnering with Shell LiveWire Top Ten Innovators to create new award categories around disruptive energy and sustainable supply chain ideas that will help uncover the brightest and most innovative solutions for waste reduction, as well as enable a lower CO2 future.	Climate change is real, renewables are part of the future energy mix. But provoking a sudden death of fossil fuels isn’t plausible #IPWeek	Shell (@Shell)
RT @Shell_UKLtd: How can we be sure that the projects we support to protect and restore natural ecosystems actually deliver the results	UK Telegraph article highlights Shell, call for more action on oil theft in Nigeria, the Niger Delta‚ real tragedy	Shell (@Shell)
We are proud to share a commitment to #sustainability with @SolidiaCO2 as they develop more #sustainable concrete	RT @PPouyanne: The #Covid19 pandemic, the #Oil crisis #Climate change 3 major crises, distinct and yet connected, that we must	TotalEnergies (@TotalEnergies)
To learn more on this new partnership, you can read our press release,	#Elgin #gas #leak: #Total prepares operations to stop the leak	TotalEnergies (@TotalEnergies)

**Fig. 1:**
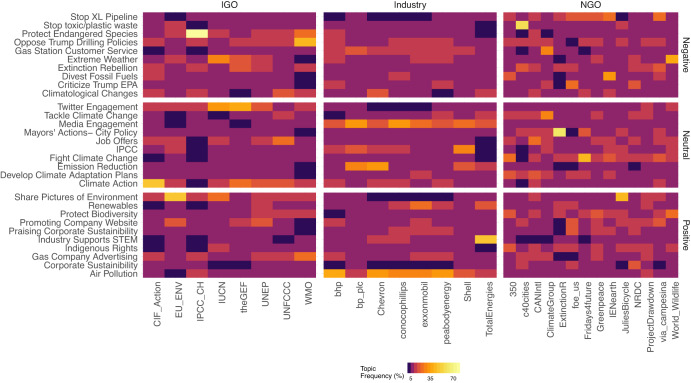
Sentiment-topic frequency distribution across the groups. A heatmap illustrating the distribution of high-frequency sentiment-topics for the social medial communications of the 8 fossil fuel firms (industry), 8 intergovernmental organizations (IGO), and 14 non-governmental organizations (NGO). Details of the tweet username and top five associated words are presented in Supplementary Tables 1 and 2, respectively. This heatmap was created using the ggplot2 v3.4.1 in the R programming language v4.1.3, further details about the dataset are in the “Data and method” section. CIF_Action Climate Innovation Fund, EU_ENV European Union Environment, IPCC_CH Intergovernmental Panel on Climate Change, IUCN International Union for Conservation of Nature, theGEF Global Environment Fund, UNEP United Nations Environment Program, UNFCCC United Nations Framework Convention on Climate Change, WMO World Meteorological Organization, bhp BHP Billiton Limited, Australia, bp_plc British Petroleum Company PLC, United Kingdom, Chevron Chevron Corporation, United States, conocophilips ConocoPhillips Company, United States, exxonmobil ExxonMobil Corporation, United States, peabodyenergy Peabody Energy, United States, Shell Shell PLC, United Kingdom, TotalEnergies TotalEnergies SE, France, 350 350.org, c40cities C40 Cities, CANIntl Climate Action Network International, ClimateGroup Climate Group, ExtinctionR Extinction Rebellion, foe_us Friend of Earth, IENearth International Indigenous Network, JuliesBicycle Julies Bicycle, NRDC Natural Resources Defence Council, ProjectDrawdown Project Drawdown, World_Wildlife World Wildlife Fund, Fridays4future Fridays for Future, Greenpeace Greenpeace, Via_campesina La Via Campesina, IGO Intergovernmental Organization, Industry Fossil fuel firms, and NGO Non-governmental Organization.

Similarly, Fig. 1 shows the common topics and their sentiments (positive, negative, and neutral) in the Twitter conversations between the fossil fuel firms (industry), IGOs, and NGOs. In particular, Fig. 1 shows the probability an organization discusses a specific topic during the entire sample period. The figure illustrates several critical differences in the frequency that these organizations discussed on Twitter regarding the topics uncovered by our model. In doing so, we note relative homogeneity in the topics about which fossil fuel firms and IGOs tweet. In contrast, NGOs are more heterogeneous on the topics they discuss on Twitter.

We now discuss topic frequencies for each group of organizations with embedded sentiments. We observe, first, fossil fuel firms post tweets that promote their outside media and public engagement with a neutral tone. This includes tweeting about news segments and newspaper articles that positively affect the firms. They are also more likely to tweet about actions taken to mitigate air pollution with a positive tone (see Fig. 1 and Table 2). Second, IGOs tend to post tweets that promote further Twitter engagement and discuss climate action, both with a neutral sentiment. They are also more likely to post about their opposition to the Trump administration’s drilling policies and protecting endangered wildlife, carrying a more negative tone. Third, NGOs, many of which represent activist groups, are more heterogeneous in their Twitter discussion topics. There are few discernible group-wide patterns, except they tend to engage evenly across the entire portfolio of topics. The topic distribution in low-dimensional space is further illustrated in Supplementary Table 8.

### Fossil fuel firms’ position in the online communications space

**Fig. 2:**
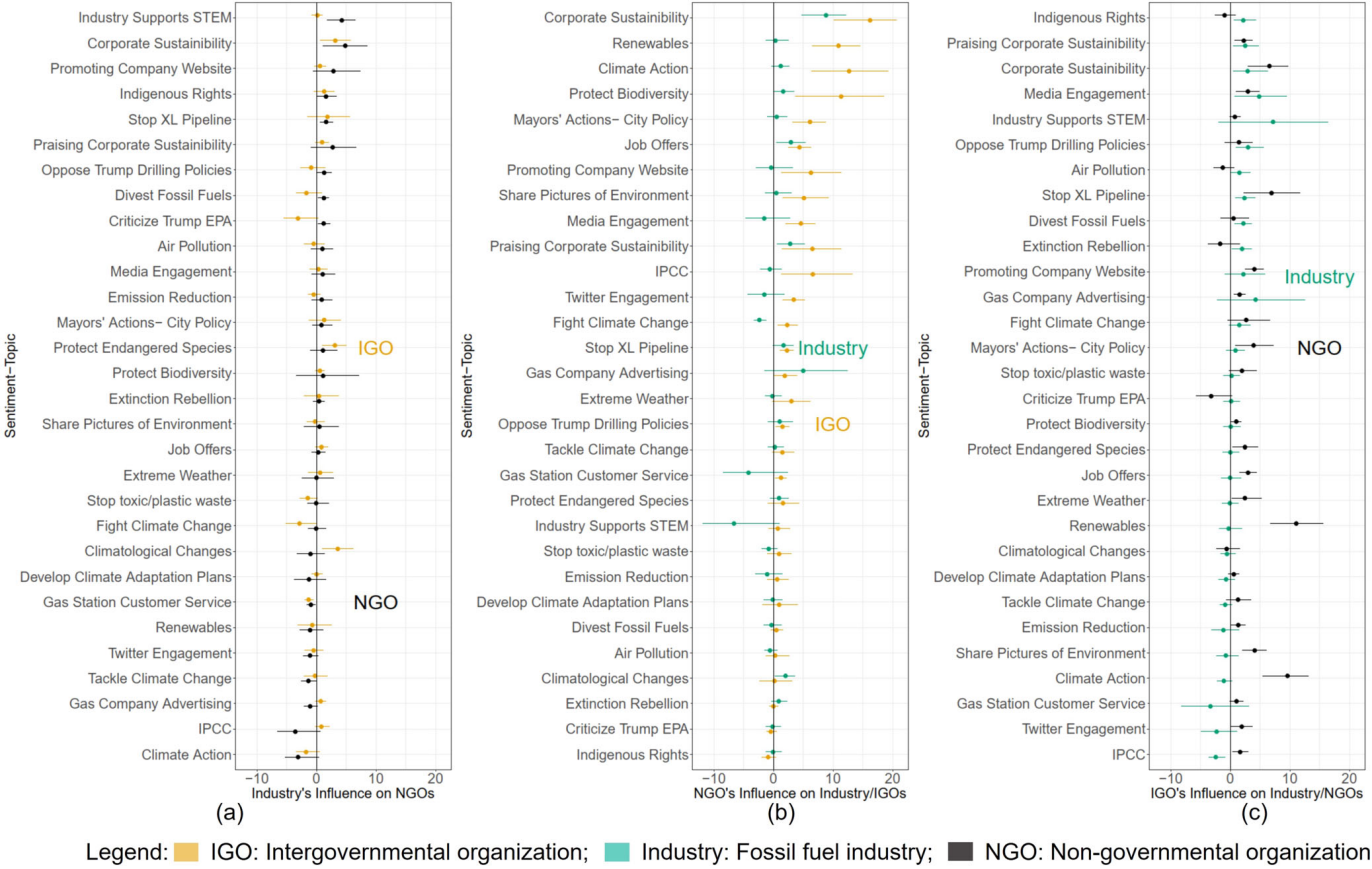
Estimated stakeholders’ influence on climate and sustainability topics. Impulse response functions (IRFs) for sentiment-topic predicted in the industry–IGO–NGO online communication space, **a** the predicted response by NGOs and IGOs to fossil fuel firms, **b** the predicted response by fossil fuel firms and IGOs to NGOs, and **c** the predicted response by fossil fuel firms and NGOs to IGOs. Bootstrapped 95 percent confidence intervals (CI) are provided. This shows the predicted change in the probability of discussing the sentiment topics discussed by fossil fuel firms, IGO, and NGOs in response to a standard deviation change of each entity of those discussing a specific topic. The results where bootstrapped 95-percent CI are statistically significant in the first period after the initial impulse is shown. Error bars show measurement uncertainty with 95-percent Bootstrapped CIs. Additional information on the daily propensity of this topic discussion is provided in Supplementary Fig. 4 and Supplementary Section 3. IGO Intergovernmental Organization, Industry Fossil fuel firms, and NGO Non-governmental Organization.

Figure 2 shows the impulse response functions (IRF) that estimate the hypothetical increase in an organization’s likelihood of discussing a topic in the face of a standard deviation increase in another organization’s average topical propensity. These results illustrate the following observations with respect to the study’s hypotheses:

*Hypothesis 1:* IRF results in Fig. 2a are consistent with the hypothesis that these organizations interact online. For instance, Fig. 2a illustrates the industry’s influence on the online communication of non-governmental organizations. The figure depicts the responsiveness of IGOs and NGOs to a hypothetical increase in fossil fuel firms’ social media communication on the topics uncovered by our JST model. Note that 30 percent of the IRFs are significant for IGOs’ response to a hypothetical increase in fossil fuel firms’ discussing a topic.

Similarly, 13 percent of IRFs are statistically significant for IGOs’ response to a hypothetical increase in fossil fuel firms’ discussing a topic. A hypothetical standard deviation increase in fossil fuel firms’ probability of discussing how they support STEM initiatives and corporate sustainability results in a 5 percent increase in NGOs’ average probability of discussing these topics, both significant at the 95 percent level. Stopping the XL pipeline, opposing Trump drilling policies, divesting from fossil fuels, and criticism of the Trump EPA all result in smaller 1 percent increases, significant at the 95 percent level. These IRF estimates are hypothetical responses, so they do not imply fossil fuel firms engaged regularly in these (often controversial) topics. To account for this, our IRF estimates are scaled by the empirical base rate for the probability that a group of organizations discussed this topic. Similarly, IGOs tend to respond to the fossil fuel industry by increasing their average propensity to discuss corporate sustainability and climatological changes by 3 percent, significant at the 95 percent level.

Figure 2b demonstrates the impact of NGOs on the industry’ and IGO’ communication. The figure depicts the responsiveness of fossil fuel firms and IGOs to a hypothetical increase in NGOs’ social media communication on the topics uncovered by our joint sentiment-topic model. Note that 57 percent of the IRFs are significant for IGOs’ response to a hypothetical increase in NGOs’ discussing a topic. Similarly, 20 percent of IRFs are statistically significant for fossil fuel firms’ response to a hypothetical rise in NGOs’ debating an issue. Results indicate a significant impact on online IGO communication over biodiversity protection; for example, the IGO stakeholder group is ∼11 percentage points more inclined to debate this topic.

Similarly, IGO correspondence is more likely to address issues related to governance and city policies (see ‘Mayor’s action—City Policy’ in Fig. 2b) in neutral sentiment. Similarly, the IGO stakeholder group is ∼14 percentage points more likely to affect NGO communication on climate action subjects (with neutral sentiment). Renewable energy (positive sentiment), employment opportunities (neutral sentiment), corporate sustainability (positive), and media interaction (neutral) are likely to influence an NGO’s Twitter engagement by 12–16 percentage points.

Figure 2c depicts the responsiveness of fossil fuel firms and NGOs to a hypothetical increase in IGOs’ social media communication on the topics uncovered by our joint sentiment-topic model. Note that 53 percent of the IRFs are significant for NGOs’ response to a hypothetical increase in IGOs discussing a topic. Similarly, 33 percent of IRFs are statistically significant for fossil fuel firms’ response to a hypothetical rise in IGOs discussing a topic. If the results were purely noise, this rate exceeds the 5 percent one might expect. Findings indicate that the industry is likelier to interact with IGOs on media engagement and advertising-related themes. For example, topics such as industry support for STEM (∼8 percentage points in positive sentiment) and gas company advertising (∼5 percentage points in positive sentiment). Similarly, there is a notable influence of NGOs on IGOs’ Twitter communication, specifically on climate action (∼10 percentage points in neutral sentiment) and renewable energy-related topics (∼12 percentage points in positive sentiment, see Fig. 2c).

*Hypothesis 2:* Taken together, Fig. 2 helps to show the interactions of all three groups in the online communication space. Relatively large effect sizes in Fig. 2a relative to Fig. 2b and c would be evidence consistent with this hypothesis. Whereas we expected fossil fuel firms, a powerful interest group with billions of dollars in resources, to be relatively more influential than NGOs and IGOs in the online discussion space, we find the opposite. Based on our evidence from the IRF, we find that IGOs and NGOs are responsive when fossil fuel firms increase their discussion on a selected number of topics, most strongly for corporate sustainability and when the industry supports STEM initiatives. Yet, at the same time, we find that IGOs are responsive to nongovernmental actors (mainly activist groups) in the same way, especially for corporate sustainability and climate action. Fossil fuel firms are also responsive, particularly for corporate sustainability and praise for corporate sustainability initiatives. Finally, NGOs are responsive to hypothetical increases in IGOs’ probability of discussing corporate sustainability, stopping the XL pipeline, and renewables. The industry also responds to hypothetical increases on some topics but with smaller magnitudes. They are most likely to respond to hypothetical increases in IGOs’ probability of discussing corporate sustainability and praising corporate advertising and media engagement on sustainability.

### Other factors affecting fossil fuel firms’ communication

**Fig. 3:**
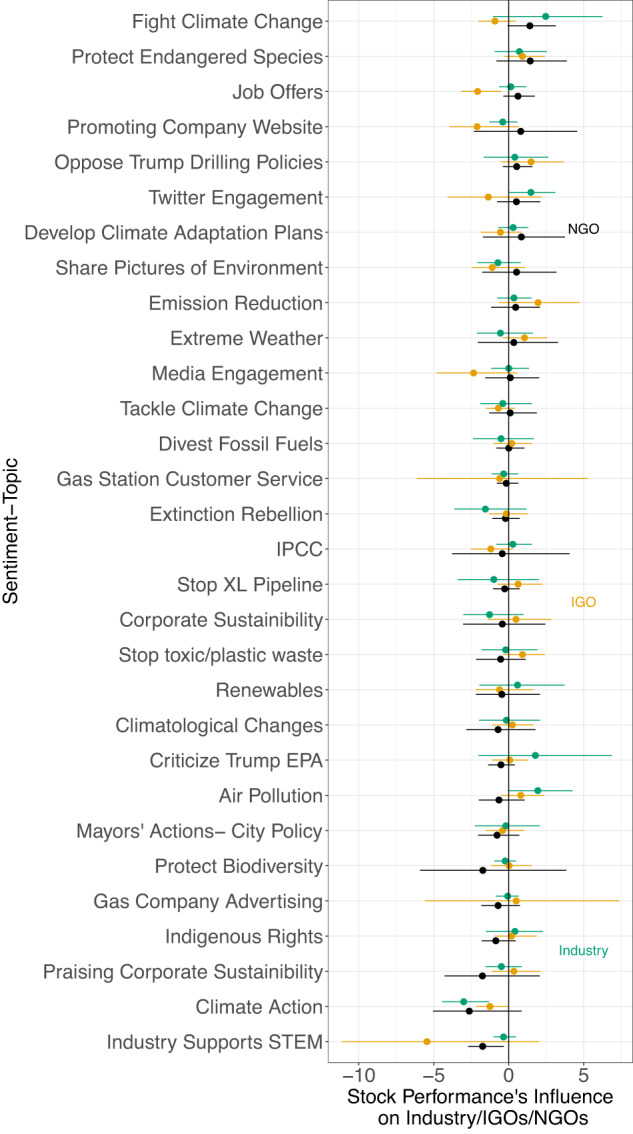
Influence of stock market performance on stakeholders’ online communication. Estimated impulse response functions (IRFs) for sentiment-topics associated with a positive standard deviation shock to large fossil fuel firms’ average stock market returns. Bootstrapped 95 percent confidence intervals are shown. The results where the 95 percent CI is statistically significant in the first period are shown. Error bars show measurement uncertainty with 95-percent Bootstrapped CIs. The average daily stock market returns are shown in Supplementary Fig. 5 with summary statistics in Supplementary Table 4. IGO Intergovernmental Organization, Industry fossil fuel firms, and NGO non-governmental organization.

**Fig. 4:**
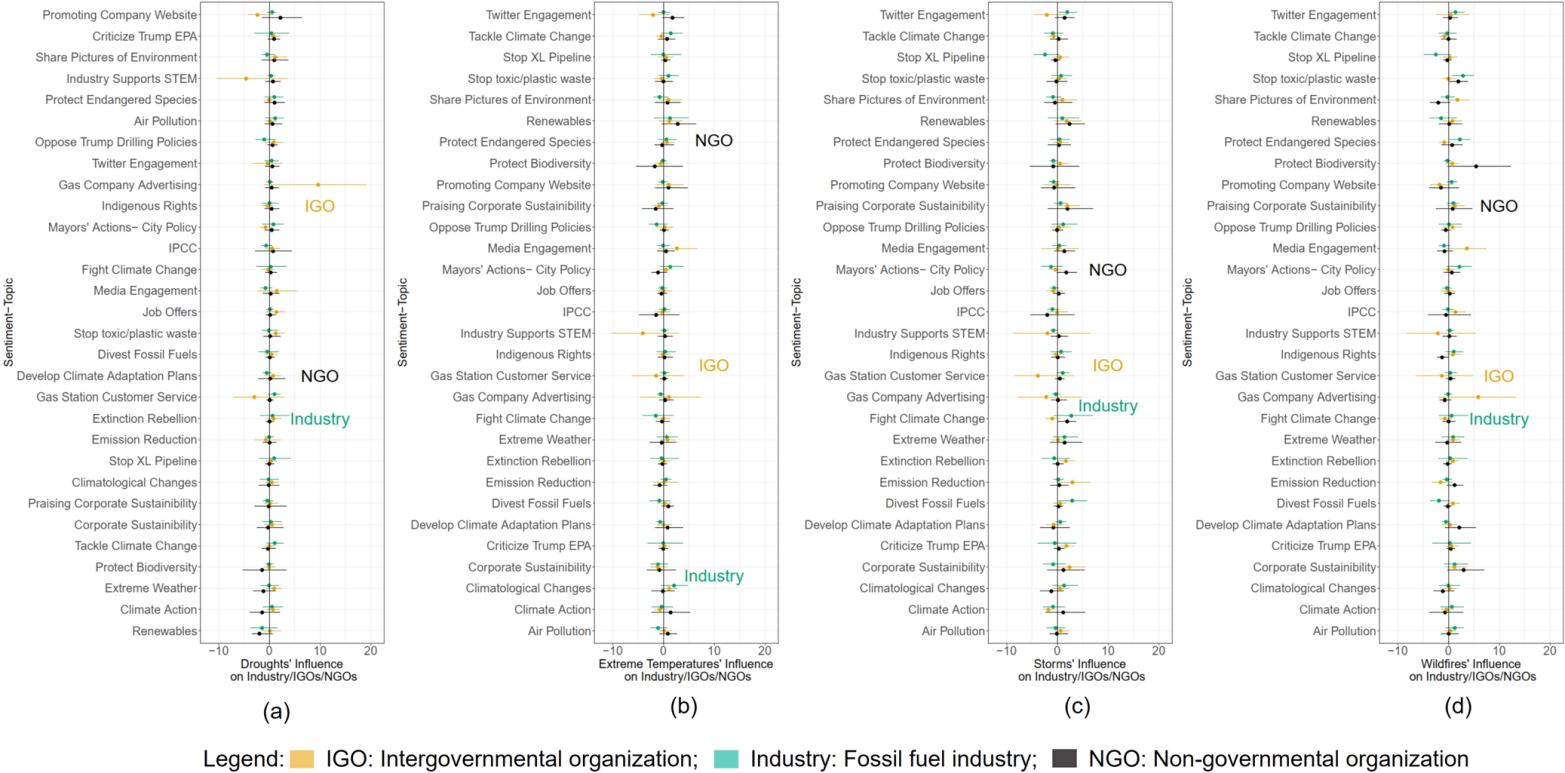
Influence of extreme weather events on stakeholders’ online communication. Estimated impulse response functions for sentiment-topics associated with selected extreme weather events based on EM-DAT’s publicly available weather datasets (2014–2021): **a** Drought, **b** Extreme temperature, **c** Storm and **d** wildfires. Bootstrapped 95 percent confidence intervals are shown. The results where the 95 percent CI is statistically significant in the first period are shown. Error bars show measurement uncertainty with 95-percent Bootstrapped Cis. IGO Intergovernmental Organization, Industry fossil fuel firms, and NGO non-governmental organization.

*Hypothesis 3:* Here, we expected extreme weather events to have a negligible effect on online messaging because social media is global and weather events tend to be localized, acute phenomena. We also expected that fossil fuel firms’ online communications would be correlated with stock performance, as these returns represent their bottom line (consistent with refs. ^38,40^). Null effects for weather events in Fig. 4 and statistically significant effects on fossil fuel firms’ coefficients in Fig. 3 provide evidence consistent with this hypothesis.

Results show neither factor is strongly correlated with online communication. In our VAR framework, we treat stock returns for the fossil fuel firms in our sample as an additional endogenous variable while treating weather events as exogenous controls. Both are accounted for in the results reported in Fig. 2. To denote weather events, we use the EM-DAT database (see the section “Data and method”) of disasters to mark days where drought, extreme temperatures, storms, and wildfires caused over 1 million inflation-adjusted dollars in damage during the sample period. Moreover, Fig. 3 shows that IGOs’ and NGOs’ strategic communication decisions did not respond directly to the top fossil fuel firms’ stock market returns. We tested for potential changes at the 95 percent level. The IRF analysis shows that fossil fuel firms, IGOs, and NGOs did not change their messaging based on daily average stock returns.

Next, we examine the responsiveness of these organizations to extreme weather events on their propensity to discuss specific topics. The IRF analysis included controls for extreme weather events like drought, extreme temperature, storms, and wildfires on industry-IGO-NGO communication, shown in Fig. 4. Results for drought (Fig. 4a) show null or weak effects across all sentiment topics except a strong influence on the IGOs’ discussion on gas company advertising (10 percentage points). Similarly, in the case of extreme temperature, the IRF analysis showed no or weak effects across all topics by the stakeholders (see Fig. 4b).

The IRF results for the association between extreme storms and stakeholders’ online messaging also show null or weak effects across all sentiment-topics, except IGO-led topics associated with gas station customer service (∼7 percentage points) and industry’s messaging on company advertising (∼4.5 percentage points, see Fig. 4c). Similarly, wildfires did not influence the industry–IGO–NGO online communication for most sentiment-topics with significant effects. However, a significant effect was seen across NGOs’ propensity to discuss topics related to the protection of biodiversity (5 percentage points) and the industry’s greater propensity to discuss gas company advertising by 5.5 percentage points (see Fig. 4d). Thus, fossil fuel firms’ advertising and public relations are highly likely online messaging topics when stakeholders discuss extreme weather events.

## Discussion

In this study, we analyzed the online social media communication structures of the top eight largest greenhouse gas-emitting firms in the fossil fuel industry, 14 non-governmental organizations (NGOs), and eight inter-governmental organizations (IGOs) with a total of 9.6 million followers worldwide. We tested three hypotheses related to the nature of the online interactions of these stakeholders concerning climate change and sustainability messaging. First, utilizing natural language processing-driven joint sentiment-topic (JST) modeling and vector autoregression (VAR), we empirically analyzed the influence of industry–IGOs–NGOs on sustainability and climate-related themes on Twitter between 2014 and 2021 (*n* = 668,826). Moreover, the VAR results demonstrated that the directionality of online communication is neither influenced by the industry’s stock performance nor impacted by extreme weather occurrences such as storms, drought, wildfires, and high temperatures.

We used JST modeling to reduce the dimensionality of our text dataset to 30 discernible sentiment topics, built from 668,826 tweets. We find substantial discussion amongst fossil fuel firms, IGOs, and NGOs regarding sustainability and climate issues (see Fig. 1). Yet, climatological changes, criticism of Trump’s drilling and environmental protection policies, divestment from fossil fuels, extreme weather, and gas station customer service were mentioned at least 70% of the time in the online communication space of the industry, with negative sentiment embeddings. Similarly, the most prevalent negative sentiment topics (with more than 85% repetition) among the IGOs’ tweets were related to the conservation of endangered species, resistance to Trump’s drilling policy, and extreme weather events.

At least 80% of the NGOs’ online communication characterized by negative sentiments were around stopping toxic and plastic waste, extreme weather, fossil fuel divestment, and Trump’s environmental protection initiatives (see Fig. 1). Therefore, we revealed a structural distinction between industry, IGOs, and NGOs regarding online social media communication themes.

The impulse response function (IRF) analysis from the VAR model illustrates the influence of fossil fuel firms on IGOs and NGOs’ (and vice versa) social media communication at 95% confidence levels. We found that climate stakeholders influence other stakeholders within their respective topical domains. When fossil fuel firms increase their online discussion of corporate sustainability and corporate STEM initiative-related topics, NGOs are predicted to be ∼8 percentage points more likely to discuss it over the following week (see Fig. 2a). Consistent with our hypotheses (*Hypothesis 1*), our analysis offers observational evidence that the top polluting fossil fuel firms are responsive to online communication by NGOs on topics associated with environmental justice and climate change themes. IGOs were the most responsive group, with predicted increases of 10–20 percentage points in their probability of discussing topics related to climate action if NGOs increase their discussion of those topics.

In response to IGOs, Fig. 2c shows that fossil fuel firms and NGOs are responsive to policy issues, such as promoting renewables, stopping the XL pipeline, and opposing the Trump administration’s drilling policies. NGOs are particularly responsive to increases in IGOs’ propensity to discuss climate action initiatives. These findings are consistent with our first hypothesis. Furthermore, this finding dovetails with Supran and Oreskes’s^13^ analysis of ExxonMobil’s climate communication. They found that the company had publicly overemphasized (a reframing behavior) some terms and topics associated with greenwashing while avoiding others as a form of climate misinformation.

Contrary to *Hypothesis 2*, NGOs have more sway over online conversations according to our VAR framework. Despite being less well-funded and with fewer institutional levers of power, these groups can influence the online discourse on climate change and sustainability. Moreover, they can do so more than fossil fuel firms and intergovernmental organizations. This finding can be due to a greater number of Twitter followers associated with NGO user accounts.

Our findings related to *Hypothesis 3* are mixed. Beyond evidence for the null correlations between extreme weather events and online discourse, we also show in Fig. 3 that stock returns are largely uncorrelated with online communications. Not surprisingly, IGOs and NGOs are unmoved by stock market performance. Still, it is surprising there is no discernible relationship for fossil fuel firms since maximizing financial performance is one of a private corporation’s primary objectives. There are a few possible explanations: first, the time series data contain latent relations not captured by our VAR models. We find this unconvincing, as our analysis allows for a highly flexible time series structure, including lags. This null evidence leads us to believe that even if there is a relationship, it is likely to be of a small magnitude. A second and more plausible explanation is that corporate public relations strategies are driven by long-term messaging goals unrelated to the company’s actual financial performance. This explanation suggests how the fossil fuel industry might capitalize on green narratives for climate action and sustainability, ultimately avoiding messages directly linked to their financial situation (also reported by refs. ^14,36,38,40,42^).

Our paper contributes to the growing literature on climate accountability by advancing the understanding of “reframing” by influential stakeholders on social media platforms, as they aim to influence online climate and sustainability communications. We envisage countering such subtle climate communication reframing through regulatory and design changes on social media platforms, which would enable them to be more exhaustive and transparent. In addition, self-regulation and community engagement around such reframing behavior might motivate behavioral design changes^34,44^ to support individuals’ assessment of the information they access—a vital element to generate consensus around climate action.

This paper suggests future opportunities to explore whether these macro-level discourses between core stakeholder groups lead to opinion or behavior change. Future work also involves the development of data-driven frameworks to derive a typology of the structure of climate misinformation based on how influential stakeholders communicate on online platforms. Future research could also examine the data-driven value chain of climate and sustainability information that feeds these stakeholder groups.

Our study has potential limitations. For instance, lexicon-based sentiment analysis has limitations in capturing the true language meaning and small changes if the words are changed due to their annotation and coding definitions. This remains an open challenge with discriminative NLP models. In addition, Twitter may be subject to demographic biases. In this paper, we limited such biases by analyzing the entire Twitter corpus of the concerned organizations to capture the more extensive cross-sectional breadth of online communication and embedded valence shifters in the tweets’ semantic structure. We also limit biases using weakly supervised and unsupervised learning methods (like the JST), which require limited human intervention and training data. Furthermore, we validated our extracted sentiment-topic scores using multiple human readers to reduce lexicon-based discrepancies. Other methods can be used to reduce the dimensionality of social media conversations, for example, the structural topic model, large-language models, and transformer-based approaches. As we have shown, weakly supervised approaches like the JST can identify topics and produce sensible results without utilizing potentially biased and expensive human-labeled training data. Whether neural network-based large-language and generative AI models might be useful is an area for future research. A final limitation regards our assumption that a greater number of followers on Twitter means a more significant influence on the communication that motivated the selection of fossil fuel firms, IGOs, and NGOs.

Our observational research shows that social media data helps study industry-level behavior on climate change and sustainability and its propensity to influence the communication of other crucial stakeholder groups, potentially affecting consensus around effective climate action. In sum, our paper provides a new direction for understanding the structure of the online climate and sustainability information ecosystem using social media big data.

## Data and method

To empirically consider our hypotheses, we use natural language processing (NLP) in a new way to measure information flows in stakeholders’ climate communications on social media. First, we use the joint sentiment-topic (JST) methodology to determine the topical structure of the social media conversation between the three-climate stakeholder groups^45,46^. Then, we take the topical proportions uncovered by the JST model and employ dimensional reduction to quantify and visualize the discussion space for these stakeholders, examine interactions in their communication, and identify the triggers that influence the reframing of online communication (see Supplementary Section 2).

With the results of the NLP modeling, we then investigate who leads and who follows in social media debates about specific climate and sustainability topics. We examine the primary topics of IGOs and NGOs and their sensitivity to the industry’s propensity to drive the conversation using vector autoregression (VAR). We control for considerations exogenous to the communication environment, such as extreme weather events and the industry’s stock market performance. Using impulse response functions (IRF) calculated from the VAR model (see Supplementary Section 3), we establish quantifiable associations between climate-related communication among large fossil-fuel firms, IGOs, and NGOs. These methodological steps are discussed below, with additional supporting details in the Supplementary Information. For details on the vars v1.5-9 package used to implement the time series analysis in R, please refer to refs. ^47^^,48^.

This research was reviewed by the Institutional Review Board at the Judge Business School, University of Cambridge (20-064) and at the California Institute of Technology (21-1169). Twitter was informed about this research during the v2API request.

### Data source

We use the Twitter (now known as X) v2API endpoints to collect daily time-series tweets for the stakeholder groups shown in Supplementary Table 1 between January 2014 to September 2021, accessed using the academictwitterR v0.3.0 package^49^ in R-programming language. As a search query, we used the username of the official global Twitter accounts of the organizations (see Supplementary Table 1), as they had the largest follower counts and most tweets were in the English language to reach maximum users.

More specifically, in the “industry” category, we included investor-owned top polluting fossil fuel firms in this study^43,50^. The global spread of firms contains BP (UK), Chevron (USA), BHP (Australia), ConocoPhillips (USA), ExxonMobil (USA), Peabody Energy (USA), Shell (The Netherlands) and Total Energies (France). Cumulatively, these firms emitted ∼207,361 million tonnes of carbon dioxide equivalent (MtCO_2_e) between 1965 and 2018 (Table 1).

We curated the list of environmental and intergovernmental (IGOs) and non-governmental organizations (NGOs) based on organizational size using the publicly available University of California Berkeley’s database^51^ and DonorBox’s 2021 classification of top environmental protection non-profits from their portfolio of more than 50,000 organizations in 96 countries^52^. In the next step, we manually filtered the NGOs and IGOs list based on two criteria: (a) English-language Twitter user accounts that have at least 10,000 followers, and (b) availability of seven years (January 2014–September 2021) of time-series historical tweets. Our final dataset contained a cumulative follower base of 9.6 million users worldwide and 728,967 tweets. Detailed descriptive characteristics of our dataset are presented in Supplementary Tables 1 and 2. We acknowledge that the Twitter dataset may contain some unintentional biases. For example, our data may contain tweets from malicious users artificially causing a topic or a hashtag to trend, or tweets that misrepresent already trending items using bots^53^. We follow established best practice guidelines to mitigating such biases^54,55^.

The daily stock market return data are the calculated (or derived) firm equity prices based on the daily stock market returns data from the CRSP US Stock Database ©2021, provided by the Centre for Research in Security Prices (CRSP), The University of Chicago Booth School of Business. In addition, stock market returns summary statistics are provided in Supplementary Table 4 with average daily returns in Supplementary Fig. 5. The extreme weather dataset is extracted from EM-DAT (https://www.emdat.be). It is a database of global disasters maintained by the Centre for Research on the Epidemiology of Disasters (CRED) at the School of Public Health of the Université Catholique de Louvain located in Brussels, Belgium, maintaining records of historical global disasters from 1900 of meteorological, epidemiological, and other natural origin.

### Pre-processing

We follow best practices for pre-processing text-as-data^56–58^. To ensure we have the right number of features; we stem the tokens that will enter the JST bag-of-words model. For valid inference from text data, researchers should strive for both enough common words such that there is shared variation amongst documents. At the same time, they must balance this against the need for enough unique words that are not shared amongst many documents so that distinct topics can be identified by the topic model.

We take the following steps to create a list of 1009 unique words in our dataset, which has 330,412 tweets post-pre-processing:

1. Drop all tweets that do not contain three unique words.
2. Stem all words to remove word endings to distill core semantic meaning.
3. Find bigrams and trigrams and tokenize.
4. We drop all words that appear in fewer than 0.005 percent of tweets in the corpus and in more than 50 percent of tweets in the corpus, respectively, following the advice in ref. ^56^.

The tweets were processed with an NLP workflow (following refs. ^25,59^) using the tidyverse v1.3.1 and tidytext v0.3.2 packages in R. The workflow consisted of text pre-processing, feature extraction for n-grams, and sentiment analysis. The pre-processing stage consisted of tokenisation, stemming, and lemmatisation. In NLP, tokenisation refers to breaking down the given text into smaller units in a sentence called token^60,61^. Stemming in NLP is a morphological technique that breaks words into their root form^61^. Finally, lemmatisation is another normalization technique used to reduce inflectional forms of words to a common base form^61^. It differs from stemming as it uses lexical knowledge bases to get the correct base forms of words^61^. At this NLP pre-processing stage, we removed the stopwords using the tm v0.7-8 package in R. Stopwords are the most common words in any language (like articles, prepositions, pronouns, conjunctions, etc.) that do not add much information to the text. For example, common stopwords in English are “the”, “a”, “an”, “so”, and “what”^61^. This workflow extracted the cleaned base form of words and generated a document-term-matrix (dtm) needed for further analysis.

The average tweet length in our corpus is 18.31. Post-processing, the average unique tokens per tweet is 17.31 with 1009 unique words. The total number of unique tweets per stakeholder group is 57,550 for industry, 50,059 for IGOs, and 222,813 for NGOs. We had an unbalanced selection of organizations in the NGO category (14) compared to eight each for the fossil industry and IGOs, leading to more unique tweets.

As an initial exploratory text analysis, all tweets were analyzed using the sentimentr v0.4.0 package in R to estimate the text polarity sentiments at the sentence level and optionally aggregate by rows or grouping variable(s). sentimentr is an augmented dictionary lookup tool which attempts to embed valence shifters (i.e., negators, amplifiers (intensifiers), de-amplifiers (downtoners), and adversative conjunctions) in the NLP-driven sentiment analysis while maintaining computation speed^62^.

Valence shifters affect the polarized words. For example, in the case of negators and adversative conjunctions, the entire sentiment of the clause may be reversed or overruled^63^. The tweet corpus analyzed here is from the global corporate accounts of the fossil fuel firms, IGOs and NGOs, so accounting for valence shifts was important to appropriately estimate the embedded sentiments.

### Joint sentiment-topic modeling (JST)

To understand the nature of online discussion between IGOs, NGOs, and fossil fuel firms, we employ a topic modeling method to uncover the rates at which the organizations in our dataset discuss topics. The method both uncovers the topics and the probabilities that each document belongs to each of the topics. We know that online communication might have sentimental inflection (certain words have different meanings in different contexts), so we employ a topic model that accounts for this sentiment, Joint-Sentiment Topic Modeling (JST). Detailed modeling specification is shown in Supplementary Section 2 JST Evaluation.

The JST model is similar to the Latent Dirichlet Allocation algorithm for topic modeling, except that JST estimates topics *k* conditional on a sentiment *j*. The JST model thus estimates three latent layers (sentiment orientation, topic classification, and word probabilities belonging to both)^45,46^, which provides a critical methodological advantage when assessing interactive semantic exchanges, like Twitter messaging interactions across multiple stakeholders. It is a Bayesian hierarchical mixture model with three hyperparameters *α*, *β,* and *γ*.

The hyperparameter α is the prior concentration of the sentiment-topic *k*_*i*_ for a document before having seen any documents from the corpus. Similarly, hyperparameter *β* is the prior concentration of the sentiment-topic *j* for a word before any words from the corpus are observed. And the hyperparameter γ is the prior concentration of the sentiment labels sampled under a document before having seen any documents.

In our model, we estimated the unconditional probability of each sentiment *j* as a weakly supervised model by placing a weak prior over the sentiment orientations for a selection of common words. This approach captured the entire discussion space of the industry, IGOs, and NGOs on Twitter without relying on exogenous covariates to uncover the latent space. Therefore, enabling us to model Twitter communication at a systems level and examine the industry-IGO-NGO systemic interactions.

A critical feature of the semantic structure extracted by the JST is the distinct variation in how industry, IGOs, and NGOs communicate on social media, even when projected into a lower dimensional space. Therefore, we produced a probability distribution for every word and each of the 728,967 tweets in the dataset, which can be decomposed as Eq. (1).

1 $$\begin{array}{ll}\,\,\,\,{\rm {Pr}}({\rm {Word}}=w,{\rm {Sentiment}}=j,{\rm {Topic}}=k)\\ \qquad\qquad\,\,\,\,\,={\rm {Pr}}({\rm {Word}}=w|{\rm {Sentiment}}=j,{\rm {Topic}}=k)\\ \,\ast {\rm {Pr}}({\rm {Topic}}=k|{\rm {Sentiment}}=j)\end{array}$$

This produced a vector of *kj* sentiment-topic probabilities and *j* sentiment probabilities for each tweet so that the sentiment-topic labels are independent.

In our analysis, we followed standard practice and set a relatively small prior with the assumption that the tweets, given their concise nature (maximum character limit is 280), are likely to relate to very few topics at once^45^. Therefore, as *β* goes to 0, the model converges to a model of a single sentiment-topic. Furthermore, the limiting distribution gets uniform over the sentiment-topic as *β* grows larger.

As mentioned above, we calibrate the model by optimizing the coherence score that suggests the optimal number of topics is 30 (see Supplementary Fig. 2). In addition, we set the number of sentiments to 3 (sent1, sent2, and sent3) following the paradigmatic prior in ref. ^45^. It resulted in 180 conditional sentiment-topic probabilities and three unconditional sentiment probabilities for each tweet. We further illustrated the top 30 topics using sentiment-topic occurrence frequencies (Fig. 1) and used the rJST v1.3 package and default rJST dictionary for constructing the JST model. Additional supporting details regarding the JST analysis are provided in Supplementary Section 2, including an extracted corpus of the top 5 topic words (see Supplementary Table 2) and probabilistic topic labeling (emblematic) (Supplementary Table 3, emblematic tweets). The topic proportions per information source are presented in Supplementary Table 2.

### Vector autoregression (VAR)

Our dynamic analysis of the Twitter sentiment-topics from the fossil fuel firms, the NGOs, and the IGOs, was conducted using vector autoregression (VAR). First, we evaluated the probabilities of IGOs and NGOs to discuss each sentiment-topic in their daily Twitter communications. We use an impulse response framework from the time series VAR of topical probabilities derived from our JST estimates. Building on a methodological framework used in previous research^64^, our estimation strategy estimates a VAR for each topic, treating each organization’s average probability of discussing that topic and fossil fuel firms’ stock performance, as well as a topic discussion under the influence of extreme weather events as endogenous, using publicly available EM-DAT database.

VAR is a time series technique that allows for the analysis of how a particular time-series of data responds to its history and the history of other time-series in the analysis, which has been used in previous studies that examine the dynamics of multiple time-series of Twitter topics^64^. VAR also allows us to simulate how changes in one time series (“shocks”) may work through the entire system of equations. As our data are stationary but censored between 0 and 1 as in ref. ^64^, we follow’s ref. ^65^ logit specification for VAR. Supporting details about the VAR analysis are provided in Supplementary Section 33, and specifically, Supplementary Fig. 3 provides details about stationarity.

Supplementary Section 3 contains more details about our time series analysis and robustness check performed using Augmented Dickey–Fuller Tests for Unit Root (shown in Supplementary Fig. 3). In addition, it includes summary statistics for the daily propensity of fossil firms’ Twitter activity on the primary topic in the analysis (Supplementary Table 5). Similarly, Supplementary Tables 6 and 7 show summary statistics for IGOs and NGOs topic time series, respectively. The daily propensity of the stakeholders to discuss divestment from fossil fuels, renewable jobs, and extreme weather is shown in Supplementary Fig. 4. The average daily stock return is shown in Supplementary Fig. 4d, with stock market returns summary statistics presented in Supplementary Table 4.

Using this topic-by-topic model, we measure the online information ecosystem of the stakeholder groups in our dataset and data from exogenous weather events, such as droughts, wildfires, storms, and extreme temperatures. Using these model estimates, we compute impulse response functions (IRFs) for the probability each type of organization discusses a topic when another group increases its average probability of discussing it. Specifically, these IRFs estimate the hypothetical increase in an organization’s likelihood of discussing a topic in the face of a standard deviation increase in another organization’s average topical propensity.

### Reporting summary

Further information on research design is available in the Nature Research Reporting Summary linked to this article.

### Supplementary information

Reporting Summary

Supplementary information

## Data Availability

The materials necessary to reproduce the results reported in this paper are available at https://github.com/danielEban ks/. Energy-Industry-Greenwashing. Per the terms of Twitter’s academic use policies, we will make available the tweet IDs for the data used in this paper upon publication. Researchers can obtain the CRSP data from https://www.crsp.org/. Publicly available extreme weather events data can be obtained from EM-DAT (https://www.emdat.be). Alternatively, please contact the corresponding author to request the dataset.

## Code availability

The code necessary to reproduce the results reported in this paper is available at the following GitHub repository: https://github.com/danielEbanks/Energy-Industry-Greenwashing.

## References

[1] IPCC. *Climate Change 2022: Mitigation of Climate Change. Contribution of Working Group III to the Sixth Assessment Report of the Intergovernmental Panel on Climate Change* (Cambridge University Press, 2022).

[2] Turrentine, J. *Climate Misinformation On Social Media Is Undermining Climate Action* (NRDC, 2022).

[3] Debnath, R., Creutzig, F., Sovacool, B. K. & Shuckburgh, E. Harnessing human and machine intelligence for planetary-level climate action. *npj Clim. Action* **2**, 10.1038/s44168-023-00056-3 (2023). 10.1038/s44168-023-00056-3 PMC11062317 38694954

[4] Rathje, S., Van Bavel, J. J. & van der Linden, S. Out-group animosity drives engagement on social media. *Proc. Natl. Acad. Sci*. *USA* **118**, 10.1073/pnas.2024292118 (2021). 10.1073/pnas.2024292118 PMC8256037 34162706

[5] Treen, K. M. D., Williams, H. T. P. & O’Neill, S. J. Online misinformation about climate change. *WIREs Clim. Chang*. **11**, 10.1002/wcc.665 (2020).

[6] Wetts R (2020). In climate news, statements from large businesses and opponents of climate action receive heightened visibility. Proc. Natl. Acad. Sci. USA.

[7] Supran G, Oreskes N (2017). Assessing ExxonMobil’s climate change communications (1977–2014). Environ. Res. Lett..

[8] Li, M., Trencher, G. & Asuka, J. The clean energy claims of BP, Chevron, ExxonMobil and Shell: a mismatch between discourse, actions and investments. *PLoS ONE* **17**, 10.1371/journal.pone.0263596 (2022). 10.1371/journal.pone.0263596 PMC8849545 35171938

[9] Pérez-González L (2020). ‘Is climate science taking over the science?’: a corpus-based study of competing stances on bias, dogma and expertise in the blogosphere. Humanit. Soc. Sci. Commun..

[10] Brevini, B. & Lewis, J. *Climate Change and the Media* Vol. 2 (Academic Publishers, 2018).

[11] Feldman L, Hart PS, Milosevic T (2017). Polarizing news? representations of threat and efficacy in leading US newspapers’ coverage of climate change. Public Underst. Sci..

[12] CCDH. *The Toxic Ten: How Ten Fringe Publishers Fuel 69% of Digital Climate Change Denial* (CCDH, 2021).

[13] Supran G, Oreskes N (2021). Rhetoric and frame analysis of ExxonMobil’s climate change communications. One Earth.

[14] Lewton T, McCool A (2021). Greenwashing on facebook: how the world’s biggest polluters use social media to obfuscate on climate change. Time.

[15] Supran, G., Rahmstorf, S. & Oreskes, N. Assessing Exxonmobil’s global warming projections. *Science* 379, 10.1126/science.abk0063 (2023). 10.1126/science.abk0063 36634176

[16] Farrell J (2016). Network structure and influence of the climate change counter-movement. Nat. Clim. Chang..

[17] Boussalis C, Coan TG (2016). Text-mining the signals of climate change doubt. Glob. Environ. Chang..

[18] Micheals, D. *Doubt is Their Product: How Industry’s Assault on Science Threatens Your Health* (Oxford University Press, 2008).

[19] Cinelli M, De Francisci Morales G, Galeazzi A, Quattrociocchi W, Starnini M (2021). The echo chamber effect on social media. Proc. Natl. Acad. Sci. USA.

[20] Farrell J (2019). The growth of climate change misinformation in us philanthropy: evidence from natural language processing. Environ. Res. Lett..

[21] Ranganathan, N. & Beltrán, M. eco-bot.net. Dale Vince, Robert '3D' Del Naja & Bill Posters (2021).

[22] UCS. *Smoke, Mirrors & Hot Air—How ExxonMobil Uses Big Tobacco’s Tactics to Manufacture Uncertainty on Climate Science* (UCS, 2007).

[23] Fownes, J. R., Yu, C. & Margolin, D. B. Twitter and climate change. *Sociol. Compass* **12**, 10.1111/soc4.12587 (2018).

[24] Pearce, W., Niederer, S., Özkula, S. M. & Sánchez Querubín, N. The social media life of climate change: platforms, publics, and future imaginaries. *WIREs Clim. Chang*. **10**, 10.1002/wcc.569 (2019).

[25] Debnath R (2023). Conspiracy spillovers and geoengineering. iScience.

[26] Lewandowsky S, Ecker UK, Cook J (2017). Beyond misinformation: understanding and coping with the “post-truth” era. J. Appl. Res. Mem. Cogn..

[27] Dunlap, R., McCright, A., Dryzek, J., Norgaard, R. & Schlosberg, D. *The Oxford Handbook of Climate Change and Society.* Oxford University Press, Oxford, UK (2011).

[28] Dunlap RE (2013). Climate change skepticism and denial. Am. Behav. Sci..

[29] Törnberg, P. Echo chambers and viral misinformation: Modeling fake news as complex contagion. *PLoS ONE* **13**, 10.1371/journal.pone.0203958 (2018). 10.1371/journal.pone.0203958 PMC6147442 30235239

[30] Cook J, Ellerton P, Kinkead D (2018). Deconstructing climate misinformation to identify reasoning errors. Environ. Res. Lett..

[31] Linden SVD, Leiserowitz A, Rosenthal S, Maibach E (2017). Inoculating the public against misinformation about climate change. Glob. Challenges.

[32] Cook, J., Lewandowsky, S. & Ecker, U. K. H. Neutralizing misinformation through inoculation: Exposing misleading argumentation techniques reduces their influence. *PLoS ONE* **12**, 10.1371/journal.pone.0175799 (2017). 10.1371/journal.pone.0175799 PMC5419564 28475576

[33] Kyza, E. A. et al. Combating misinformation online: re-imagining social media for policy-making. *Internet Policy Rev*. **9**, 10.14763/2020.4.1514 (2020).

[34] UNICEF. *Countering Online Misinformation Resource Pack* (UNICEF, 2020).

[35] Debnath R, van der Linden S, Alvarez RM, Sovacool BK (2023). Facilitating system-level behavioural climate action using computational social science. Nat. Hum. Behav..

[36] Esty, D. C. & Bell, M. L. Business leadership in global climate change responses. *Am. J. Public Health* **108**, 10.2105/ajph.2018.304336 (2018). 10.2105/AJPH.2018.304336 PMC5922211 29698101

[37] Smith SR, Christie I (2021). Knowledge integration in the politics and policy of rapid transitions to net zero carbon: a typology and mapping method for climate actors in the UK. Sustainability.

[38] Cano-Marin E, Mora-Cantallops M, Sánchez-Alonso S (2023). Twitter as a predictive system: a systematic literature review. J. Bus. Res..

[39] Bollen J, Mao H, Zeng X (2011). Twitter mood predicts the stock market. J. Comput. Sci..

[40] Oliveira N, Cortez P, Areal N (2017). The impact of microblogging data for stock market prediction: using Twitter to predict returns, volatility, trading volume and survey sentiment indices. Expert Syst. Appl..

[41] Kirilenko AP, Molodtsova T, Stepchenkova SO (2015). People as sensors: Mass media and local temperature influence climate change discussion on Twitter. Glob. Environ. Chang..

[42] Dellmuth, L. & Shyrokykh, K. Climate change on Twitter: implications for climate governance research. *WIREs Clim. Chang*. 10.1002/wcc.848 (2023).

[43] Kenner D, Heede R (2021). White knights, or horsemen of the apocalypse? prospects for big oil to align emissions with a 1.5c pathway. Energy Res. Soc. Sci..

[44] Lorenz-Spreen P, Lewandowsky S, Sunstein CR, Hertwig R (2020). How behavioural sciences can promote truth, autonomy and democratic discourse online. Nat. Hum. Behav..

[45] Lin, C. & He, Y. Joint sentiment/topic model for sentiment analysis. In *Proc. 18th ACM Conference on Information and Knowledge Management—CIKM ’09* (eds Chueng, D., Song, I-Y., Chu, W., Hu, X. & Lin, J.) (Association for Computing Machinery, New York, NY, United States, 2009).

[46] Lin C, He Y, Everson R, Ruger S (2012). Weakly supervised joint sentiment-topic detection from text. IEEE Trans. Knowl. Data Eng..

[47] Pfaff B (2008). Var, svar and svec models: implementation within R package vars. J. Stat. Softw.

[48] Pfaff, B. *Analysis of Integrated and Cointegrated Time Series with R* 2nd edn (New York, Springer, 2008).

[49] Barrie C, Ho J (2021). Academictwitter: an R package to access the Twitter academic research product track V2 API endpoint. J. Open Source Softw..

[50] Taylor, M. & Watts, J. *Revealed: the 20 Firms Behind a Third of All Carbon Emissions* (2019).

[51] Berkeley Library, University of California. Non-governmental organizations (NGOs). University of California Berkeley, CA, United States (2022).

[52] Raviraj. *Top 23 Global Nonprofits Protecting the Environment*. Donorbox, VA, United States (2022).

[53] Cook D, Waugh B, Abdipanah M, Hashemi O, Rahman S (2014). Twitter deception and influence: issues of identity, slacktivism, and puppetry. J. Inf. Warf..

[54] Al Baghal, T., Wenz, A., Sloan, L. & Jessop, C. Linking Twitter and survey data: asymmetry in quantity and its impact. *EPJ Data Sci*. **10**, 10.1140/epjds/s13688-021-00286-7 (2021).

[55] Morstatter F, Liu H (2017). Discovering, assessing, and mitigating data bias in social media. Online Soc. Netw. Media.

[56] King G, Hopkins D (2010). Extracting systematic social science meaning from text. Am. J. Political Sci..

[57] Grimmer J, Stewart BM (2013). Text as data: the promise and pitfalls of automatic content analysis methods for political texts. Political Anal..

[58] Denny MJ, Spirling A (2018). Text preprocessing for unsupervised learning: why it matters, when it misleads, and what to do about it. Political Anal..

[59] Debnath, R. et al. Social media enables people-centric climate action in the hard-to-decarbonise building sector. *Sci. Rep.* **12**, 10.1038/s41598-022-23624-9 (2022). 10.1038/s41598-022-23624-9 PMC9671910 36396727

[60] Grefenstette, G. Tokenization. In Ide, N., Véronis, J. & van Halteren, H. (eds) *Syntactic Wordclass Tagging* Vol. 9, *Text, Speech and Language Technology* 117–133 (Springer Netherlands, Dordrecht, 1999).

[61] Manning, C. D., Raghavan, P. & Schutze, H. *Introduction to Information Retrieval* (Cambridge University Press, Cambridge, 2008).

[62] Rinker, T. & Spinu, V. Trinker/sentimentr: Version 0.4.0 (2016). CRAN

[63] Yu, H., Shang, J., Hsu, M., Castellanos M. & Han, J. Data-driven contextual valence shifter quantification for multi-theme sentiment analysis. In *Proc. 25th ACM International Conference on Information Knowledge Management* (eds Mukhopadhyay S, & Zhai, C.) (Association for Computing Machinery, New York, NY, United States, 2016). 10.1145/2983323.2983793 PMC5319421 28232874

[64] Barbera P (2019). Who leads? Who follows? Measuring issue attention and agenda setting by legislators and the mass public using social media data. Am. Political Sci. Rev..

[65] Wallis, K. F. Time series analysis of bounded economic variables. *J. Time Ser. Anal.* **8**, 115–123 (1987).

