# Supplementary material for: Do fossil fuel firms reframe online climate and sustainability communication? A data-driven analysis
Source: NPJ Clim Action. 2023 Dec 18;2(1):47. doi: 10.1038/s44168-023-00086-x (PMC11062293; doi:10.1038/s44168-023-00086-x)
Supplement: Supplementary file 1 — Reporting Summary [file 44168_2023_86_MOESM1_ESM.pdf]

nature portfolio

Corresponding author(s): Ramit Debnath

Last updated by author(s): Oct 4, 2023

Reporting Summary

Nature Portfolio wishes to improve the reproducibility of the work that we publish. This form provides structure for consistency and transparency in reporting. For further information on Nature Portfolio policies, see our Editorial Policies and the Editorial Policy Checklist.

Statistics

For all statistical analyses, confirm that the following items are present in the figure legend, table legend, main text, or Methods section.

n/a	Confirmed	
☐	☒	The exact sample size (*n*) for each experimental group/condition, given as a discrete number and unit of measurement
☐	☒	A statement on whether measurements were taken from distinct samples or whether the same sample was measured repeatedly
☐	☒	The statistical test(s) used AND whether they are one- or two-sided<br>*Only common tests should be described solely by name; describe more complex techniques in the Methods section.*
☐	☒	A description of all covariates tested
☐	☒	A description of any assumptions or corrections, such as tests of normality and adjustment for multiple comparisons
☐	☒	A full description of the statistical parameters including central tendency (e.g. means) or other basic estimates (e.g. regression coefficient) AND variation (e.g. standard deviation) or associated estimates of uncertainty (e.g. confidence intervals)
☐	☒	For null hypothesis testing, the test statistic (e.g. *F*, *t*, *r*) with confidence intervals, effect sizes, degrees of freedom and *P* value noted<br>*Give P values as exact values whenever suitable.*
☒	☐	For Bayesian analysis, information on the choice of priors and Markov chain Monte Carlo settings
☒	☐	For hierarchical and complex designs, identification of the appropriate level for tests and full reporting of outcomes
☒	☐	Estimates of effect sizes (e.g. Cohen's *d*, Pearson's *r*), indicating how they were calculated

Our web collection on statistics for biologists contains articles on many of the points above.

Software and code

Policy information about availability of computer code

Data collection	Twitter (now known as X) v2API endpoints to collect daily time-series tweets between January 2014 to September 2021, accessed using academictwitterR v0.3.0. Stock market data was provided by CRSP US Stock Database ©2021, provided by the Center for Research in Security Prices (CRSP), The University of Chicago Booth School of Business. The extreme weather dataset is extracted from EM-DAT (https://www.emdat.be). It is a database of global disasters maintained by the Centre for Research on the Epidemiology of Disasters (CRED) at the School of Public Health of the Université Catholique de Louvain located in Brussels, Belgium, maintaining records of historical global disasters from 1900 of meteorological, epidemiological, and other natural origin.
Data analysis	All natural language processing and econometric data analysis was performed in R RStudio 2023.03.0+386 for macOS, using following packages: sentimentr v0.4.0, tidyverse v1.3.1, tidytext v0.3.2, tm v0.7-8, rJST v1.3, vars v1.5-9.<br>All codes are accessible here: https://github.com/danielEbanks/Energy-Industry-Greenwashing

For manuscripts utilizing custom algorithms or software that are central to the research but not yet described in published literature, software must be made available to editors and reviewers. We strongly encourage code deposition in a community repository (e.g. GitHub). See the Nature Portfolio guidelines for submitting code & software for further information.

Data

Policy information about availability of data

All manuscripts must include a data availability statement. This statement should provide the following information, where applicable:

- Accession codes, unique identifiers, or web links for publicly available datasets
- A description of any restrictions on data availability
- For clinical datasets or third party data, please ensure that the statement adheres to our policy

Per the terms of Twitter's academic use policies, we will make available the tweet IDs for the data used in this paper upon publication. Researchers can obtain the CRSP data from https://www.crsp.org/. Publicly available extreme weather events data can be obtained from EM-DAT (https://www.emdat.be). Alternatively, please contact the corresponding author to request the dataset.

Research involving human participants, their data, or biological material

Policy information about studies with human participants or human data. See also policy information about sex, gender (identity/presentation), and sexual orientation and race, ethnicity and racism.

Reporting on sex and gender	NA
Reporting on race, ethnicity, or other socially relevant groupings	NA
Population characteristics	NA
Recruitment	NA
Ethics oversight	This research was reviewed by the Institutional Review Board at the Judge Business School, University of Cambridge (20-064) and at the California Institute of Technology (21-1169). Twitter was informed about this research during the v2API request.

Note that full information on the approval of the study protocol must also be provided in the manuscript.

Field-specific reporting

Please select the one below that is the best fit for your research. If you are not sure, read the appropriate sections before making your selection.

☐ Life sciences ☒ Behavioural & social sciences ☐ Ecological, evolutionary & environmental sciences

For a reference copy of the document with all sections, see nature.com/documents/nr-reporting-summary-flat.pdf

Behavioural & social sciences study design

All studies must disclose on these points even when the disclosure is negative.

Study description	Mixed-method study design involving natural language processing, sentiment analysis and vector autoregression.
Research sample	668,826 tweets, daily stock market data from CRSP US Stock Database, daily extreme weather data from EM-DAT.
Sampling strategy	Dynamic tweets were collected using v2API that provided about 1% of all Tweets in real-time. Only global English-language accounts of the stakeholder groups were targeted to stream tweets, using its Twitter username.
Data collection	Data collected using Twitter v2API endpoints to collect daily time-series tweets between January 2014 to September 2021, accessed using academictwitterR v0.3.0.
Timing	January 2014 to September 2021
Data exclusions	No data was excluded from the analysis
Non-participation	NA
Randomization	Randomization provided by v2API that provided about 1% of all Tweets in real-time.

Reporting for specific materials, systems and methods

We require information from authors about some types of materials, experimental systems and methods used in many studies. Here, indicate whether each material, system or method listed is relevant to your study. If you are not sure if a list item applies to your research, read the appropriate section before selecting a response.

Materials & experimental systems

n/a	Involved in the study
☒	☐ Antibodies
☒	☐ Eukaryotic cell lines
☒	☐ Palaeontology and archaeology
☒	☐ Animals and other organisms
☒	☐ Clinical data
☒	☐ Dual use research of concern
☒	☐ Plants

Methods

n/a	Involved in the study
☒	☐ ChIP-seq
☒	☐ Flow cytometry
☒	☐ MRI-based neuroimaging
