# Supplementary material for: Do fossil fuel firms reframe online climate and sustainability communication? A data-driven analysis
Source: NPJ Clim Action. 2023 Dec 18;2(1):47. doi: 10.1038/s44168-023-00086-x (PMC11062293; doi:10.1038/s44168-023-00086-x)
Supplement: Supplementary file 2 — Supplementary information [file 44168_2023_86_MOESM2_ESM.pdf]

# Supplementary Information: Do fossil fuel firms reframe online climate and sustainability communication? A data-driven analysis

Ramit Debnath[1,2,*], Danny Ebanks[2], Kamiar Mohaddes[1], Thomas Roulet[1], and R. Michael Alvarez[2]

[1]University of Cambridge, Cambridge, CB2 1TN, United Kingdom
[2]California Institute of Technology, Pasadena, 91125, United States
[*]corresponding author - rd545@cam.ac.uk

# Supplementary Section 1 Twitter Data

In Supplementary Table 1 we provide the complete list of the 8 non-governmental organizations (NGOs), the 14 intergovernmental organizations (IGOs), and the 8 fossil fuel firms (industry) and their respective follower counts (until September 2021) analyzed in our study. We used the global Twitter handles (usernames) of the organizations. References are presented in "Data Source" in the Method section of the manuscript.

Supplementary Table 1: Twitter usernames and follower count (until September 2021) of stakeholders used in this study

| Organisation | Username | Followers (in thousands) |
|---|---|---|
| Non-governmental organizations | | |
| 350.org | 350 | 393.2 |
| C40Cities | c40cities | 112.1 |
| Climate Action Network International | CANIntl | 42.1 |
| Climate Group | ClimateGroup | 156.5 |
| Extinction Rebellion | ExtinctionR | 386.8 |
| Friend of Earth | foe_us | 219.9 |
| International Indigenous network | IENearth | 82.7 |
| Julies Bicycle | JuliesBicycle | 11.4 |
| Natural Resources Defence Council | NRDC | 344.5 |
| Project Drawdown | ProjectDrawdown | 47.5 |
| World Wildlife Fund | World_Wildlife | 1400 |
| Fridays for Future | Fridays4future | 143.4 |
| Greenpeace | Greenpeace | 1800 |
| La Via Campesina | via_campesina | 25.9 |
| Intergovernmental organizations | | |
| Climate Innovation Fund | CIF_Action | 21.1 |
| EU Environment | EU_ENV | 129.80 |
| Intergovernmental Panel on Climate Change | IPCC_CH | 327.40 |
| International Union for Conservation of Nature | IUCN | 186.1 |
| Global Environment Fund | theGEF | 80.60 |
| United Nations Environment Programme | UNEP | 1100 |
| United Nations Framework Convention on Climate Change | UNFCCC | 878.7 |
| World Meteorological Organization | WMO | 145.6 |
| Fossil Industry | | |
| BHP Billiton | bhp | 56.1 |
| British Petroleum | bp_plc | 106.7 |
| Chevron | Chevron | 374.8 |
| Conoco Philips | conocophillips | 163.6 |
| ExxonMobil | exxonmobil | 328.1 |
| Peabody Energy | peabodyenergy | 8.7 |
| Total Energies | TotalEnergiesPR | 16 |
| Shell | Shell | 566.2 |
| Total followers | | 9655.5 |

Supplementary Figure 1 provides the time series of the total number of tweets per day across the stakeholder groups that we collected and analyzed in this study between 2014-2021.

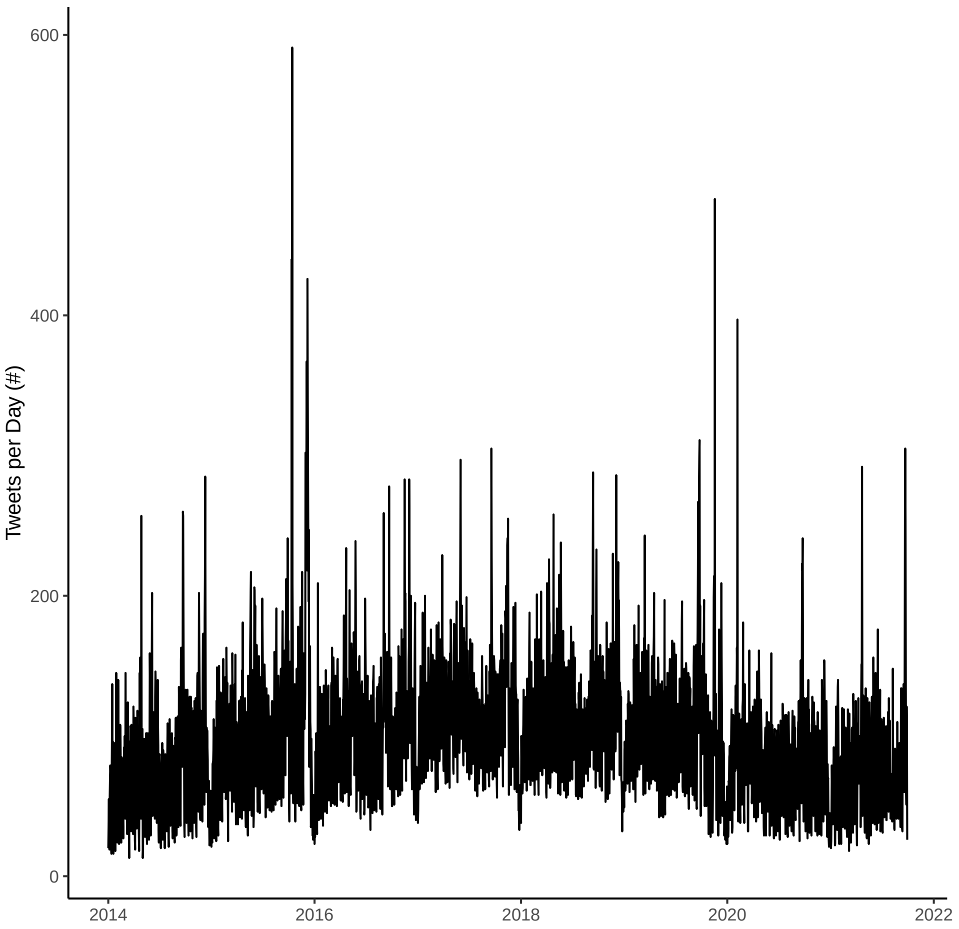


Supplementary Figure 1: Tweets Per Day

# Supplementary Section 2 JST Evaluation

In addition, our JST model relied on exchangeability and a bag-of-words approach to speech that made the model mutually exclusive to the order of the words as the sentiment-topics were discovered across the stakeholder groups. It was done to optimize the computation costs with the coherence measure of the sentiment-topic clusters. A similar optimization approach was also taken by [2, 3]. Our pseudo-code for the data-generation process for the documents is summarized as follows:

1. For each tweet $t$, choose a distribution $\pi_t \sim$ Dirichlet $(\gamma)$. Where, $pi_t$ is a multinomial distribution over sentiments for each document drawn from a Dirichlet prior.

2. For each sentiment label $j$ under tweet $t$, choose a topic distribution $\theta \sim$ Dirichlet $(\alpha)$. Where, $\theta$ is a multinomial distribution (drawn from a Dirichlet prior) over topics for each tweet conditional over a sentiment.

3. For each word $w_i$ in tweet $t$,

   (a) Choose a sentiment label $j_i$ from $pi_t$.

   (b) Choose a topic label $k_i$ from $\theta_{t,l_i}$.

   (c) Choose a word $w_i$ from the distribution, $\phi_{j_i,k_i}$ over words defined by the topic $k_i$ and sentiment $j_i$. Here, $\phi_{j_i,k_i}$ is a distribution over word given being in sentiment label $j_i$ and topic label $k_i$ under sentiment $j_i$.

As a Bayesian hierarchical mixture model, we consider the hyperparameter $\alpha$ as the prior concentration of the sentiment-topic $k_i$ for a document before having seen any documents from the corpus. Similarly, hyperparameter

$\beta$ is the prior concentration of the sentiment-topic $j$ for a word before any words from the corpus are observed. The hyperparameter $\gamma$ is the prior concentration of the sentiment labels sampled under a document before having seen any documents.

We select the number of topics based on the inflection point beyond which increases in coherence are small. Based on this criterion, we select 28 topics. To arrive at this number, we tuned the model starting from 5 topics and 10 topics increasing in increments of 10 up to 60 topics. Realizing the inflection point was between 10 topics, we selected it as $k$. Supplementary Figure 2 shows that there is an inflection point at 10 topics, for 30 sentiment-topics. Supplementary Table 2 illustrates the top 5 tokenized words for the 30 sentiment-topics. Supplementary Table 3 shows the tweet with the highest probability of being labeled as a particular topic. This tweet is emblematic in that it the the most likely tweet to exemplify the topic.

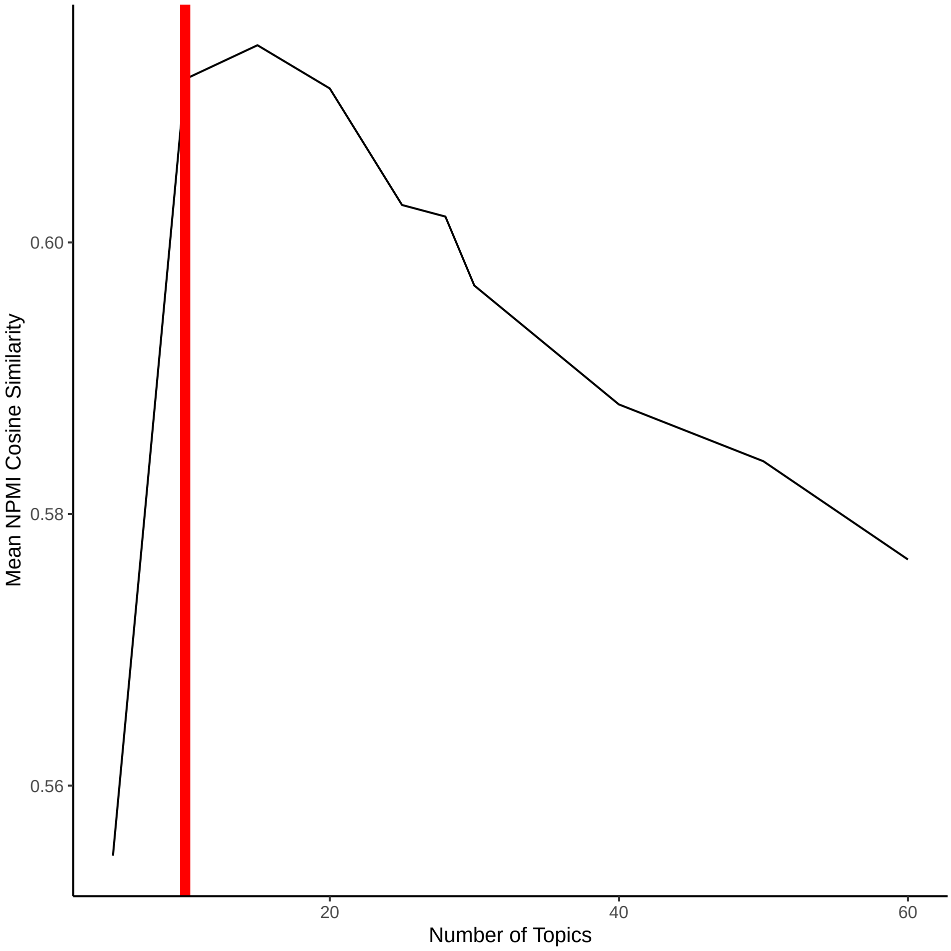


Supplementary Figure 2: Coherence by Topic

Supplementary Table 2: Top 5 Topic Words

| | 1 | 2 | 3 | 4 | 5 |
|---|---|---|---|---|---|
| Fight Climate Change | climat | action | crisi | justic | climat_crisi |
| Industry Supports STEM | chevron | support | stem | mt | team |
| Protect Endangered Species | speci | endang | wildlif | protect | save |
| Job Offers | job | offer | offr | job_offer | demploi |
| Indigenous Rights | coal | forest | indigen | right | plant |
| Gas Station Customer Service | parti | pass | contact | dm | station |
| IPCC | ipcc | climatechang | report | climat | event |
| Renewables | energi | renew | solar | power | clean |
| Stop toxic/plastic waste | food | plastic | pesticid | wast | ocean |
| Climate Action | climat | action | cop21 | parisagr | pari |
| Air Pollution | air | pollut | water | clean | air_pollut |
| Stop XL Pipeline | pipelin | nokxl | sand | keyston | tar |
| Tackle Climate Change | climat | chang | climat_chang | fight | action |
| Corporate Sustainibility | food | sustain | environ | system | futur |
| Extinction Rebellion | climat | govern | extinctionrebellion | ecolog | pm |
| Twitter Engagement | energi | carbon | electr | renew | busi |
| Protect Biodiversity | forest | biodiv | thegef | natur | ecosystem |
| Climatological Changes | global | warm | sea | record | rise |
| Media Engagement | live | watch | question | energi | tune |
| Share Pictures of Environment | day | photo | world | check | video |
| Extreme Weather | weather | extrem | flood | climat | climatechang |
| Emission Reduction | emiss | reduc | carbon | gas | co2 |
| Promoting Company Website | chevron | site | check | live | career |
| Divest Fossil Fuels | fuel | fossil | fossil_fuel | climat | industri |
| Mayors' Actions- City Policy | citi | climat | mayor | action | c40 |
| Gas Company Advertising | energi | oil | gas | bp | product |
| Oppose Trump Drilling Policies | oil | drill | land | arctic | trump |
| Develop Climate Adaptation Plans | develop | climat | countri | financ | sustain |
| Praising Corporate Sustainibility | environ | sustain | üáüá | natur | green |
| Criticize Trump EPA | trump | epa | environ | pruitt | health |

Supplementary Table 3: Emblematic Tweets

| handle | text | label |
|---|---|---|
| c40cities | Air pollution causes longlasting harm and is a national public health crisis This new data shows that the action Im taking is already making a difference and saving lives MayorofLondon is taking action to protect residents amp improve air quality | Air Pollution |
| CANIntl | I urge all countries to make the Katowice Climate Conference a success and heed the counsel of the worlds top scientists raise ambition rapidly strengthen their national climate action plans and urgently accelerate implementation of the Paris Agreement COP24 | Climate Action |
| WMO | TODAYUNSG antonioguterres speech on the State of The Planet and WMO StateofClimate report underline the need for more ClimateAction WMO report gives details on temperatures ocean heat sea level rise ice melt extreme weather etc in 2020ClimateChange ParisAgreement | Climatological Changes |
| foe_us | Were working for solutions to climate change For clean energy and a safe amp healthy food system To protect forests and Indigenous communitiesYour support strengthens our commitment to defend the Earth today and everyday Help us continue the fight | Corporate Sustainibility |
| foe_us | Our environment is under threat from the pesticide industry Big Oil amp other giant corporations and Trumps administration keeps putting corporate profits ahead of people and planet Help us fight to stop Trump without spending a dime | Criticize Trump EPA |
| UNFCCC | Latest reviews of developed country climate policies and actions show progress toward emission reduction targets and increased efforts to support ClimateAction in developing countries Transparency of action is a key pillar of the ParisAgreement | Develop Climate Adaptation Plans |

| | | |
|---|---|---|
| foe_us | HUGE WIN The UK has announced an immediate end to public finance for fossil fuel project overseasIts time for EximBankUS USTDA to follow UKs lead and stop supporting oil amp gas projects all over the world We cannot continue bankrolling the climate crisis with tax dollars | Divest Fossil Fuels |
| NRDC | Great news Under a new law passed by New Yorks city council the citys largest buildings will reduce their greenhouse gas emissions 40 by 2030 Thats a huge deal because New Yorks buildings account for a whopping 67 of the citys carbon emissions | Emission Reduction |
| ExtinctionR | Inaction How about some action on the climate and ecological emergency governmentsRebelForLife | Extinction Rebellion |
| UNFCCC | July has had extreme weather from landslides amp flooding in Japan to heatwaves from China to North America Extreme heat amp precipitation events are increasing as a result of climatechange shares the WMO See CGTVs coverage of extreme weather in Japan | Extreme Weather |
| Greenpeace | Climate justice is gender justiceClimate justice is racial justiceClimate justice is economic justiceClimate justice is environmental justiceGo to to join the next global climate strike and raise your voice for climate justice UprootTheSystem | Fight Climate Change |
| bp_plc | thejamestay Hi James thanks for your comments Natural gas is the smart partner to renewables as it burns 50 cleaner than coal in power generation Renewables are important but only part of the story Other sources of energy like oil amp gas will be necessary to meet growing global demand | Gas Company Advertising |
| Chevron | katiepanda ensuring our customers have a clean safe and reliable experience is one of our core values please send us a dm with the station location and your contact information email phone and we will bring this to the attention of the appropriate parties for further investigation | Gas Station Customer Service |

| | | |
|---|---|---|
| Chevron | First we clearly stated our commitment to international human rights normsThis commits us to respecting human rights as set out in the United Nations Universal Declaration of Human Rights and other international human rights frameworks httpstco5pu9r9VaDR | Indigenous Rights |
| bp_plc | With internship opportunities falling due to COVID19 were proud to be able to welcome over 250 students in the US UK amp Canada to our summer internships but with a difference Were providing an online programme to support students across the globe to develop their skills | Industry Supports STEM |
| ExtinctionR | Events Events Events Events Events Events Events Events Events Events Events Events Events Events Events Events Events Events Events Events Events Events Events Events Events Events Events Events Events Events For Life | IPCC |
| TotalEnergies | Offre demploi SENIOR PROCESS SAFETY ENGINEERING DESIGN ENGINEER ENG 1016 Balikpapan httptinyurlcom323kysx jobs | Job Offers |
| c40cities | Today C40 mayors amp union leaders from around the world gathered to discuss the role of local national amp international leaders in scaling global ambition on climate action and a just amp green transition towards inclusive economies Stay tuned for exciting news tomorrow | Mayors' Actions- City Policy |
| UNFCCC | Starting tomorrow were launching a series of facebook live chats to shed light on the concept of Talanoa4ambition Check out todays teaser interview You can join the chats by posting your questions on our Facebook page | Media Engagement |
| NRDC | Many Americans will visit coastal national parks this summer But a report by NRDC and the NPCA shows that if the Trump admin follows through on their offshore drilling plans 68 coastal national parks could be at risk of an oil spill Take action | Oppose Trump Drilling Policies |

| | | |
|---|---|---|
| JuliesBicycle | Next ROCKH2020 Webinar Cultural Policy Driving Environmental Change i5th Sept Explore role of cultural professionals in cities sustainable development how cultural policy organisational amp local government drives environmental leadership | Praising Corporate Sustainibility |
| ClimateGroup | The official Climate Week NYC 2020 events program is now live For the first time ever Climate Week NYC now includes virtual events from all over the world and weve invited a global audience ClimateWeekNY | Promoting Company Website |
| UNEP | Forests provide vital functions including as guardians of fresh water sources amp biodiversity protectionUN report progress in protecting the worlds forests is at risk due to the devastating impacts of the COVID19 pandemic amp the planetary crises | Protect Biodiversity |
| ClimateGroup | Since 2012 Orsted has helped reduce the levelized cost of electricity from offshore wind by more than 60 meaning it is now cheaper to build amp operate offshore wind farms in Europe than new power plants running on coal gas or nuclear ClimateWeekNYC | Renewables |
| EU_ENV | To celebrate the 50th anniversary of EarthDay WWF invites you to create nature themed artwork amp share with ArtForEarthDay 1 Wildlife Day 2 Freshwater Day 3 One Planet Day 4 Food Day 5 Forests Day 6 Climate Day 7 Ocean Our symbolic choice today 12 | Share Pictures of Environment |
| foe_us | We cant continue to let pesticide companies poison our Earth kill bees at unprecedented rates amp destroy our food systemNY Its time to pass bradhoylmans Birds amp Bees Protection Act to ban beekilling neonic pesticides Take action to SaveTheBees | Stop toxic/plastic waste |
| 350 | 8200 people have already signed the PromiseToProtect a commitment to join a wave of peaceful resistance along the route of Keystone XL when called upon by local leaders People power gt dirty pipelines Keystone XL will never be built NoKXL | Stop XL Pipeline |

| | | |
|---|---|---|
| ClimateGroup | Across the world women are acting on climate change IWD2020We recognise that urgent action is needed now to address the global climate emergency strathearnrose Cabinet Secretary for Environment Climate Change and Land Reform ScotGov Under2Coalition | Tackle Climate Change |
| bp_plc | DoreMartin Hi Martin thanks for your comments We are committed to playing our part in helping the world transition to a low carbon future amp our strategy includes a framework for reducing emissions in our operations improving our products amp creating low carbon businesses | Twitter Engagement |

Supplementary Table 4: Stock Market Returns Data

| Mean | St.dev | Min | Q25 | Median | Q75 | Max |
|---|---|---|---|---|---|---|
| -0.04 | 2.03 | -17.86 | -0.93 | -0.001 | 0.82 | 16.77 |

# Supplementary Section 3 Time Series Analysis

## Supplementary Section 3.1 Overview of the Dynamic Nature of These Data

For our specification, fix a sentiment-topic label $k$ and group $j$. Let $x^k_{group_j,t}$, $x^k_{group_{-j},t}$, and $s_t$ denote the probability of the average IGO/NGO/fossil fuel firm, then average of all other groups, and then average daily stock returns, respectively. Let $X_t = \left(x^k_{group_j,t}, x^k_{group_{-j},t}, s_t\right)$ for all $k$. Then let

$$Z = \log\left(\frac{X}{1-X}\right)$$

Our specification thus is:

$$Z_t = c + \sum_{p=t-1}^{5} \beta_p Z_p + Y + \epsilon_p \tag{1}$$

where $c$ is a constant because the time series is stationary around a non-zero mean after taking logs, we use a time lag of 5 and $Y$ are weather controls. We then run this regression for all 30 sentiment-topics identified by our JST model. We find that across the VAR regressions, the average of the optimal number of lags selected by a Bayesian Information Criterion (BIC) average at 5. This reflects the notion that discussions on social media are often ephemeral. In our case, this corresponds to discussion effects on social media lasting generally for only a business week.

Following [1], we employ generalized impulse-response functions (IRFs) that are invariant to variable ordering (see Supplementary Section 3.4 for details). Computing confidence intervals for IRFs is analytically intractable, as they are functions of both the model coefficients and the model variance-covariance matrix. To overcome this problem, we bootstrap the full sampling distribution for the IRFs. We resample the dataset 500 times and re-fit the VAR on the resampled data to derive the sampling distribution for the IRFs. This accounts for sampling uncertainty in both the VAR coefficient estimates and the estimates for the VAR variance-covariance matrix. Because we account for these underlying sources of uncertainty when reporting the confidence intervals for the IRF results, we believe the reported IRF confidence intervals are generally conservative. Finally, we rescale the responding variables' impulse responses (which are given as changes in log odds) to changes in probability, assuming the base rate of the response is the empirical probability observed in the data. For an $n$ step-ahead response, we compute

$$\Theta^k_i(n) = \frac{\delta_{\mathrm{j}}}{\sigma^2_{\mathrm{j}}} \Sigma_\epsilon \beta$$

where $\delta$ is the size of the shock and set to $\sigma$, which is standard practice. The interpretation for this choice of delta is the predicted response for a hypothetical standard deviation increase in the impulse variable, which given the volatile nature of social media communications, is an appropriate counterfactual case to analyze; such standard deviation increases are common in the online discussion space.

Supplementary Table 5 provides summary statistics for the daily propensity of Industry's Twitter activity on the primary topics in the analysis. Similarly, Supplementary Table 6 shows similar summary statistics for the IGO topic time series, with Supplementary Table 7 showing the summary statistics for the NGO topic time series.

Supplementary Table 5: Daily Propensity: Industry

| Topic | Max | Mean | Median | Min | Q25 | Q75 | St.dev |
|---|---|---|---|---|---|---|---|
| Air Pollution | 90.19 | 4.40 | 3.57 | 0.06 | 2.14 | 5.47 | 4.36 |
| Climate Action | 53.66 | 8.66 | 8.03 | 0.09 | 6.43 | 10.00 | 4.64 |
| Climatological Changes | 78.33 | 8.52 | 7.84 | 0.08 | 6.27 | 10.00 | 4.80 |
| Corporate Sustainibility | 98.79 | 6.64 | 4.66 | 0.09 | 2.65 | 8.56 | 7.86 |
| Criticize Trump EPA | 63.46 | 8.40 | 7.81 | 0.08 | 6.32 | 10.00 | 4.23 |
| Develop Climate Adaptation Plans | 98.45 | 10.14 | 8.68 | 0.09 | 6.84 | 11.24 | 7.21 |
| Divest Fossil Fuels | 94.81 | 8.62 | 7.93 | 0.08 | 6.37 | 10.00 | 4.87 |
| Emission Reduction | 98.45 | 11.75 | 9.44 | 0.09 | 7.28 | 13.37 | 9.31 |
| Extinction Rebellion | 90.19 | 8.31 | 7.76 | 0.08 | 6.27 | 10.00 | 4.44 |
| Extreme Weather | 96.47 | 9.01 | 8.04 | 0.08 | 6.45 | 10.00 | 6.01 |
| Fight Climate Change | 97.85 | 8.36 | 7.77 | 0.09 | 6.19 | 10.00 | 5.12 |
| Gas Company Advertising | 98.91 | 25.10 | 22.79 | 0.09 | 12.31 | 33.83 | 18.05 |
| Gas Station Customer Service | 99.29 | 22.20 | 18.69 | 0.22 | 10.00 | 28.83 | 15.86 |
| Indigenous Rights | 96.47 | 4.88 | 3.75 | 0.06 | 2.22 | 5.70 | 5.60 |
| Industry Supports STEM | 98.91 | 23.56 | 21.07 | 0.06 | 11.47 | 32.18 | 17.24 |
| IPCC | 96.47 | 8.92 | 8.07 | 0.09 | 6.37 | 10.00 | 5.92 |
| Job Offers | 57.75 | 8.12 | 7.67 | 0.09 | 6.07 | 10.00 | 3.97 |
| Mayors' Actions- City Policy | 96.47 | 8.83 | 8.04 | 0.09 | 6.40 | 10.00 | 5.78 |
| Media Engagement | 99.16 | 13.58 | 10.00 | 0.09 | 7.74 | 16.06 | 10.71 |
| Oppose Trump Drilling Policies | 97.33 | 9.20 | 8.12 | 0.08 | 6.44 | 10.00 | 6.40 |
| Praising Corporate Sustainibility | 97.33 | 5.68 | 4.19 | 0.06 | 2.41 | 7.22 | 6.36 |
| Promoting Company Website | 99.09 | 10.03 | 6.72 | 0.06 | 3.45 | 12.44 | 12.14 |
| Protect Biodiversity | 97.33 | 5.17 | 3.94 | 0.06 | 2.28 | 6.30 | 5.61 |
| Protect Endangered Species | 94.81 | 8.43 | 7.80 | 0.08 | 6.28 | 10.00 | 4.71 |
| Renewables | 99.09 | 7.48 | 5.15 | 0.06 | 2.84 | 9.65 | 8.57 |
| Share Pictures of Environment | 98.64 | 7.06 | 4.89 | 0.06 | 2.66 | 8.71 | 8.70 |
| Stop toxic/plastic waste | 98.03 | 8.86 | 8.05 | 0.08 | 6.41 | 10.00 | 5.35 |
| Stop XL Pipeline | 96.47 | 8.46 | 7.79 | 0.08 | 6.28 | 10.00 | 5.25 |
| Tackle Climate Change | 98.45 | 8.46 | 7.89 | 0.09 | 6.33 | 10.00 | 5.19 |
| Twitter Engagement | 99.16 | 13.18 | 10.00 | 0.09 | 7.77 | 15.74 | 9.83 |

Supplementary Table 6: Daily Propensity: IGOs

| Topic | Max | Mean | Median | Min | Q25 | Q75 | St.dev |
|---|---|---|---|---|---|---|---|
| Air Pollution | 39.12 | 7.27 | 6.32 | 0.11 | 4.30 | 9.23 | 4.42 |
| Climate Action | 90.19 | 12.60 | 11.21 | 0.15 | 7.56 | 15.82 | 6.91 |
| Climatological Changes | 54.10 | 13.08 | 12.05 | 0.17 | 8.47 | 16.15 | 6.01 |
| Corporate Sustainibility | 64.95 | 12.60 | 11.19 | 0.13 | 7.51 | 15.83 | 7.67 |
| Criticize Trump EPA | 28.33 | 8.00 | 7.49 | 0.17 | 6.35 | 9.19 | 2.66 |
| Develop Climate Adaptation Plans | 98.64 | 16.72 | 15.59 | 0.20 | 11.20 | 20.83 | 8.20 |
| Divest Fossil Fuels | 57.07 | 7.91 | 7.38 | 0.17 | 6.26 | 8.87 | 2.96 |
| Emission Reduction | 53.93 | 9.04 | 7.86 | 0.15 | 5.88 | 11.17 | 4.69 |
| Extinction Rebellion | 35.85 | 8.18 | 7.57 | 0.17 | 6.37 | 9.40 | 2.90 |
| Extreme Weather | 64.45 | 13.79 | 12.66 | 0.17 | 9.04 | 17.07 | 6.43 |
| Fight Climate Change | 45.20 | 7.81 | 6.80 | 0.15 | 5.23 | 9.54 | 3.97 |
| Gas Company Advertising | 53.24 | 6.01 | 5.24 | 0.10 | 3.82 | 7.59 | 3.54 |
| Gas Station Customer Service | 96.47 | 7.72 | 7.32 | 0.17 | 6.21 | 8.56 | 3.02 |
| Indigenous Rights | 42.03 | 6.38 | 5.52 | 0.10 | 4.03 | 8.11 | 3.71 |
| Industry Supports STEM | 50.10 | 6.73 | 5.90 | 0.10 | 4.09 | 8.63 | 3.91 |
| IPCC | 98.20 | 14.18 | 12.13 | 0.15 | 7.90 | 17.43 | 9.04 |
| Job Offers | 38.82 | 5.89 | 5.63 | 0.15 | 4.54 | 6.79 | 2.39 |
| Mayors' Actions- City Policy | 48.81 | 8.46 | 7.43 | 0.15 | 5.62 | 10.43 | 4.19 |
| Media Engagement | 53.24 | 8.60 | 7.59 | 0.15 | 5.69 | 10.45 | 4.38 |
| Oppose Trump Drilling Policies | 98.45 | 8.01 | 7.44 | 0.24 | 6.31 | 8.98 | 3.57 |
| Praising Corporate Sustainibility | 98.20 | 14.37 | 12.96 | 0.10 | 8.85 | 18.48 | 8.29 |
| Promoting Company Website | 96.47 | 8.90 | 7.70 | 0.10 | 4.51 | 11.51 | 6.45 |
| Protect Biodiversity | 98.79 | 20.46 | 19.15 | 0.11 | 13.10 | 25.97 | 10.48 |
| Protect Endangered Species | 97.85 | 14.00 | 12.57 | 0.17 | 9.30 | 17.18 | 6.98 |
| Renewables | 51.72 | 7.93 | 6.86 | 0.10 | 4.33 | 10.09 | 5.22 |
| Share Pictures of Environment | 97.77 | 9.35 | 8.23 | 0.10 | 5.09 | 11.86 | 6.71 |
| Stop toxic/plastic waste | 90.19 | 11.68 | 10.51 | 0.17 | 7.85 | 14.02 | 5.59 |
| Stop XL Pipeline | 50.10 | 7.63 | 7.25 | 0.17 | 6.17 | 8.51 | 2.61 |
| Tackle Climate Change | 37.45 | 8.87 | 7.92 | 0.15 | 5.87 | 10.88 | 4.31 |
| Twitter Engagement | 53.93 | 7.83 | 6.86 | 0.15 | 5.26 | 9.65 | 3.82 |

Supplementary Table 7: Daily Propensity: NGOs

| Topic | Max | Mean | Median | Min | Q25 | Q75 | St.dev |
|---|---|---|---|---|---|---|---|
| Air Pollution | 42.48 | 12.18 | 11.56 | 2.87 | 9.39 | 14.17 | 4.22 |
| Climate Action | 41.41 | 10.74 | 10.01 | 3.38 | 8.13 | 12.33 | 4.01 |
| Climatological Changes | 31.34 | 9.31 | 8.67 | 1.73 | 6.77 | 11.27 | 3.65 |
| Corporate Sustainibility | 39.00 | 11.01 | 9.83 | 2.23 | 8.10 | 12.55 | 4.74 |
| Criticize Trump EPA | 66.33 | 12.36 | 10.53 | 2.57 | 7.85 | 15.08 | 6.79 |
| Develop Climate Adaptation Plans | 26.09 | 8.79 | 8.47 | 2.14 | 7.20 | 10.09 | 2.42 |
| Divest Fossil Fuels | 30.44 | 10.82 | 10.10 | 2.32 | 7.87 | 13.08 | 4.23 |
| Emission Reduction | 24.98 | 9.06 | 8.77 | 2.64 | 7.28 | 10.42 | 2.62 |
| Extinction Rebellion | 58.64 | 10.27 | 7.89 | 2.02 | 6.03 | 10.72 | 7.48 |
| Extreme Weather | 29.73 | 9.15 | 8.66 | 2.00 | 6.69 | 11.00 | 3.53 |
| Fight Climate Change | 55.24 | 13.52 | 11.95 | 2.63 | 9.29 | 16.47 | 5.98 |
| Gas Company Advertising | 22.58 | 7.95 | 7.73 | 1.61 | 6.50 | 9.18 | 2.16 |
| Gas Station Customer Service | 24.28 | 5.73 | 5.57 | 1.48 | 4.56 | 6.67 | 1.71 |
| Indigenous Rights | 43.39 | 12.22 | 11.68 | 3.13 | 9.54 | 14.40 | 3.91 |
| Industry Supports STEM | 17.37 | 7.66 | 7.48 | 2.27 | 6.28 | 8.82 | 2.02 |
| IPCC | 46.52 | 7.77 | 7.49 | 2.32 | 6.34 | 8.81 | 2.39 |
| Job Offers | 19.98 | 6.45 | 6.40 | 1.17 | 5.52 | 7.23 | 1.55 |
| Mayors' Actions- City Policy | 46.19 | 11.17 | 10.43 | 2.91 | 8.27 | 13.13 | 4.24 |
| Media Engagement | 44.53 | 8.67 | 8.19 | 1.90 | 6.92 | 9.79 | 2.87 |
| Oppose Trump Drilling Policies | 38.92 | 11.32 | 10.41 | 2.32 | 8.38 | 13.34 | 4.38 |
| Praising Corporate Sustainibility | 33.22 | 8.91 | 8.64 | 2.39 | 7.07 | 10.39 | 2.67 |
| Promoting Company Website | 28.24 | 7.54 | 7.35 | 2.23 | 6.07 | 8.75 | 2.16 |
| Protect Biodiversity | 25.14 | 8.45 | 8.23 | 1.61 | 6.83 | 9.70 | 2.45 |
| Protect Endangered Species | 30.86 | 9.39 | 8.86 | 2.23 | 7.12 | 11.04 | 3.44 |
| Renewables | 36.70 | 13.20 | 12.52 | 2.27 | 9.20 | 16.55 | 5.36 |
| Share Pictures of Environment | 49.73 | 10.86 | 10.32 | 2.99 | 8.51 | 12.58 | 3.69 |
| Stop toxic/plastic waste | 36.16 | 11.80 | 11.08 | 2.06 | 8.93 | 14.02 | 4.23 |
| Stop XL Pipeline | 64.11 | 9.84 | 8.74 | 1.48 | 6.58 | 11.57 | 5.37 |
| Tackle Climate Change | 32.88 | 12.80 | 12.45 | 3.40 | 10.18 | 14.93 | 3.80 |
| Twitter Engagement | 43.65 | 11.03 | 10.68 | 2.84 | 8.55 | 12.90 | 3.61 |

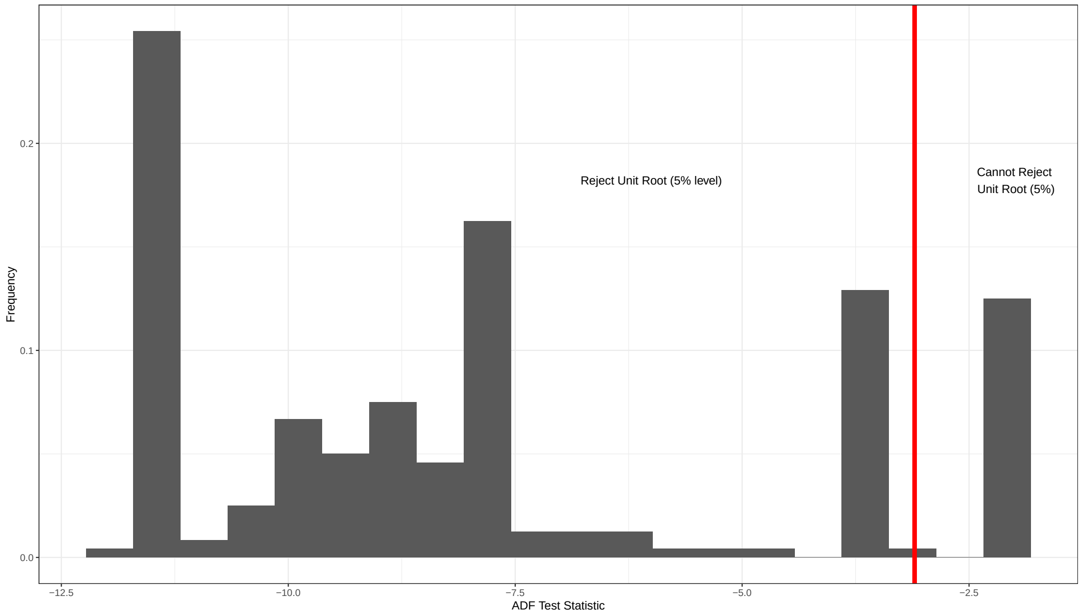


Supplementary Figure 3: Augmented Dickey-Fuller Tests for Unit Root with Trend

## Supplementary Section 3.2 Stationarity

Augmented Dickey-Fuller (ADF) test is a common statistical significance test used to test whether a given time series is stationary or not based on the presence of a unit root. The presence of a unit root means the time series is non-stationary. In Supplementary Figure 3, we provide a histogram of ADF test statistics, indicating the distribution of topics for which we reject the null of a unit root at the one percent level or at 99% significance level.

## Supplementary Section 3.3 Vector Autoregression Analysis

In Supplementary Figure 4 we provide four times series of some of the more interesting components of our VAR analysis. The remainder of these time series graphs will be available upon publication.

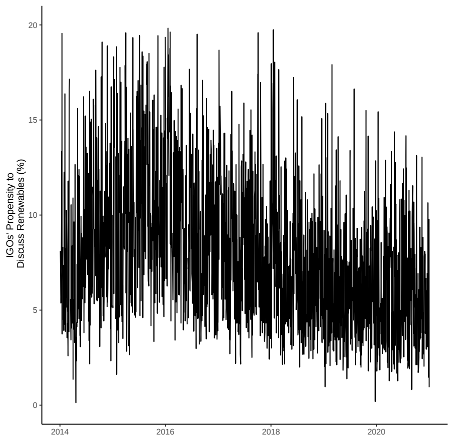


(a) Renewables - IGOs

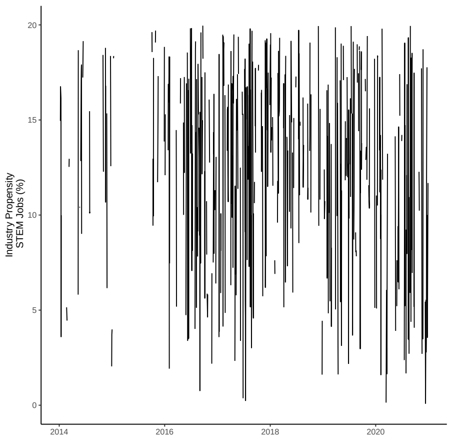


(b) STEM - Fossil Fuel Firms

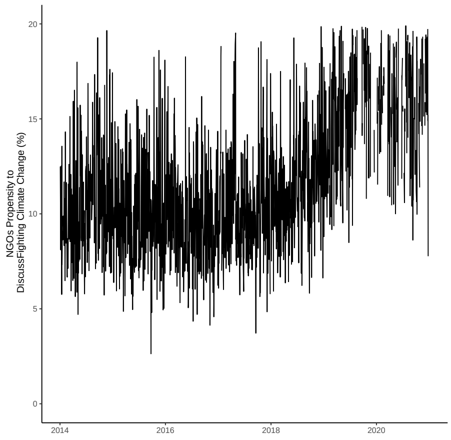


(c) Climate action - NGOs

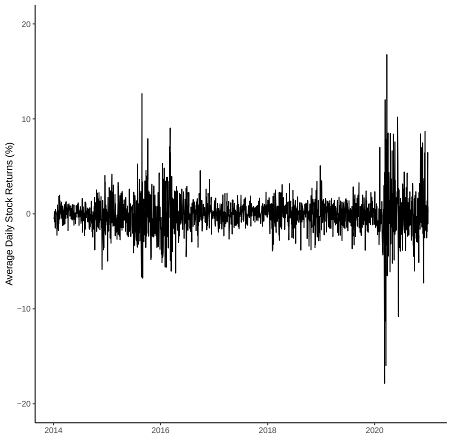


(d) Daily stock returns

Supplementary Figure 4: Daily Propensity of Discussion

### Supplementary Section 3.4 Impulse Response Function Estimation

In order to estimate the impulse response functions, we closely follow [1] and implement a version of generalized impulse response functions. Our implementation uses a modified version of the `VAR` and `irf` functions from the `vars` package in R. For details on the `var` package used to implement the time series analysis in R, please refer to [4] and [5].

We slightly modify the IRF function implemented in [5] to accommodate the generalized approach implemented in [1]. This allows for analysis of coefficients that are invariant to variable ordering. As is well known in the time-series econometric literature, IRFs are potentially sensitive to the ordering in which you analyze the variables. As we wish to compare effects across variables, we use generalized IRFs, where the results do not change depending on how one orders the variables.

To achieve this, the generalized impulse response appropriately accounts for the effects of a non-diagonal variance-covariance matrix on a potential impulse response, ie. the case when there is a estimable covariance structure in the data. Of course, it reduces to a standard IRF in the case when the off-diagonal elements of the variance-covariance matrix are 0. Unlike the orthogonalized impulse response, [1] note that the generalised IRF is unique.

The generalized impulse response we calculate is

$$GIRF = \frac{1}{\sqrt{\sigma_{ii}}} A_n \eta_i$$

where $\sigma_{ii}$ is the variance of the $i$th variable, $A_n$ is the matrix of model coefficients, and $\eta_i$ is the vector of covariances for the $i$th variable with all other variables.

## Supplementary Reference


[1] Gary Koop, M. Hashem Pesaran, and Simon M. Potter. Impulse response analysis in nonlinear multivariate models. *Journal of Econometrics*, 74(1):119–147, September 1996.

[2] Rishabh Mehrotra, Scott Sanner, Wray Buntine, and Lexing Xie. Improving LDA topic models for microblogs via tweet pooling and automatic labeling. *Proceedings of the 36th international ACM SIGIR conference on Research and development in information retrieval*, 2013.

[3] Derek O'Callaghan, Derek Greene, Joe Carthy, and Pádraig Cunningham. An analysis of the coherence of descriptors in topic modeling. *Expert Systems with Applications*, 42(13):5645–5657, 2015.

[4] B. Pfaff. *Analysis of Integrated and Cointegrated Time Series with R*. Springer, New York, second edition, 2008. ISBN 0-387-27960-1.

[5] Bernhard Pfaff. Var, svar and svec models: Implementation within R package vars. *Journal of Statistical Software*, 27(4), 2008.
